# Targeting UBC9-mediated protein hyper-SUMOylation in cystic cholangiocytes halts polycystic liver disease in experimental models

**DOI:** 10.1016/j.jhep.2020.09.010

**Published:** 2020-09-17

**Authors:** Pui Y. Lee-Law, Paula Olaizola, Francisco J. Caballero-Camino, Laura Izquierdo-Sanchez, Pedro M. Rodrigues, Alvaro Santos-Laso, Mikel Azkargorta, Felix Elortza, Maria L. Martinez-Chanta, Maria J. Perugorria, Patricia Aspichueta, Marco Marzioni, Nicholas F. LaRusso, Luis Bujanda, Joost P.H. Drenth, Jesus M. Banales

**Affiliations:** 1 Department of Liver and Gastrointestinal Diseases, Biodonostia Health Research Institute - Donostia University Hospital -, University of the Basque Country (UPV/EHU), San Sebastian, Spain; 2 Department of Gastroenterology & Hepatology, Radboud University Nijmegen Medical Center, The Netherlands; 3 National Institute for the Study of Liver and Gastrointestinal Diseases (CIBERehd, “Instituto de Salud Carlos III”), Spain; 4 Proteomics Platform, Center for Cooperative Research in Biosciences (CIC bioGUNE), ProteoRed-ISCIII, Bizkaia Science and Technology Park, Derio, Spain; 5 Liver Disease Laboratory, CIC bioGUNE, Basque Research and Technology Alliance (BRTA), Derio, Spain; 6 Department of Physiology, Faculty of Medicine and Nursing, University of Basque Country UPV/EHU, Leioa, Spain; 7 Biocruces Bizkaia Health Research Institute, Cruces University Hospital, Barakaldo, Spain; 8 “Università Politecnica delle Marche”, Department of Gastroenterology, Ancona, Italy; 9 Division of Gastroenterology and Hepatology, Mayo Clinic, Rochester, MN, USA; 10 IKERBASQUE, Basque Foundation for Science, Bilbao, Spain

**Keywords:** Hepatic cystogenesis, Post-translational modifications, SUMOylation, S-adenosylmethionine (SAMe), Therapy

## Abstract

**Background & Aims:**

Polycystic liver diseases (PLDs) are genetic disorders characterized by progressive development of multiple fluid-filled biliary cysts. Most PLD-causative genes participate in protein biogenesis and/or transport. Post-translational modifications (PTMs) are implicated in protein stability, localization and activity, contributing to human pathobiology; however, their role in PLD is unknown. Herein, we aimed to unveil the role of protein SUMOylation in PLD and its potential therapeutic targeting.

**Methods:**

Levels and functional effects of SUMOylation, along with response to S-adenosylmethionine (SAMe, inhibitor of the SUMOylation enzyme UBC9) and/or short-hairpin RNAs (shRNAs) against *UBE2I* (UBC9), were evaluated *in vitro, in vivo* and/or in patients with PLD. SUMOylated proteins were determined by immunoprecipitation and proteomic analyses by mass spectrometry.

**Results:**

Most SUMOylation-related genes were found overexpressed (mRNA) in polycystic human and rat liver tissue, as well as in cystic cholangiocytes in culture compared to controls. Increased SUMOylated protein levels were also observed in cystic human cholangiocytes in culture, which decreased after SAMe administration. Chronic treatment of polycystic (PCK: *Pkdhl*-mut) rats with SAMe halted hepatic cystogenesis and fibrosis, and reduced liver/body weight ratio and liver volume. *In vitro,* both SAMe and shRNA-mediated *UBE2I* knockdown increased apoptosis and reduced cell proliferation of cystic cholangiocytes. High-throughput proteomic analysis of SUM01-immunoprecipitated proteins in cystic cholangiocytes identified candidates involved in protein biogenesis, ciliogenesis and proteasome degradation. Accordingly, SAMe hampered proteasome hyperactivity in cystic cholangiocytes, leading to activation of the unfolded protein response and stress-related apoptosis.

**Conclusions:**

Cystic cholangiocytes exhibit increased SUMOylation of proteins involved in cell survival and proliferation, thus promoting hepatic cystogenesis. Inhibition of protein SUMOylation with SAMe halts PLD, representing a novel therapeutic strategy.

## Lay summary

Protein SUMOylation is a dynamic post-translational event implicated in numerous cellular processes. This study revealed dysregulated protein SUMOylation in polycystic liver disease, which promotes hepatic cystogenesis. Administration of S-adenosylmethionine (SAMe), a natural UBC9-dependent SUMOylation inhibitor, halted polycystic liver disease in experimental models, thus representing a potential therapeutic agent for patients.

## Graphical Abstract

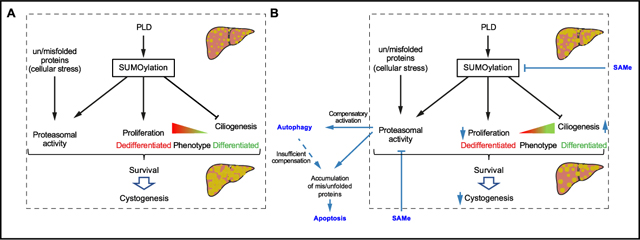

## Introduction

Polycystic liver disease (PLD) is a collection of heterogeneous genetic disorders marked by the presence of numerous fluid-filled hepatic cysts (>10) arising from the intrahepatic biliary epithelia. PLD manifests as either isolated liver cysts in autosomal dominant polycystic liver disease (ADPLD) or in conjunction with renal cysts in autosomal dominant polycystic kidney disease (ADPKD) and autosomal recessive polycystic kidney disease (ARPKD).^1^ Progressive liver cyst growth gives rise to debilitating symptoms related to organ compression, like dyspnea, early satiety and pain in the abdomen and/or back. Complications occur infrequently and consist of cyst hemorrhage, infection or rupture in cysts, as well as obstructive jaundice and portal hypertension. Current therapies include percutaneous (*i.e.,* aspiration sclerotherapy), surgical (*i.e*., liver fenestration, resection) and pharmacological (*i.e.,* somatostatin analogs) approaches, which usually show short-term and modest benefits.^1^ As a corollary, there is high demand for novel therapeutic strategies, as liver transplantation remains the only curative treatment. Unraveling the molecular mechanisms implicated in the pathogenesis of PLD aims to fulfill this unmet need.

The majority of PLD-related genes (*i.e., PRKCSH, SEC63, PKD2, GANAB, ALG8, ALG9,* and *SEC61B*) encode for endoplasmic reticulum (ER)-resident proteins involved in the synthesis, maturation, folding and transport of nascent proteins.^2^ On the other hand, mutations in the PLD-causative genes *PKD1, PKD2* and *PKHD1* directly affect proteins located into the primary cilium of cholangiocytes, leading to abnormalities in cell polarity and calcium homeostasis, which compromise multiple cell processes including protein biogenesis, proliferation, differentiation and secretion.^3^ Consequently, these gene mutations provoke global abnormalities in both cellular protein homeostasis (*i.e.,* proteostasis) and function of the protein products, resulting in ER stress and the activation of pro-survival mechanisms.^4^ Taken this knowledge into account, we hypothesized that cystic cholangiocytes might be characterized by abnormal protein dynamics linked to adaptive post-translational modification (PTM) processes, leading to the promotion of hepatic cystogenesis. Among the different PTMs, protein SUMOylation has drawn increasing attention as it is an essential and reversible process, governing a plethora of cellular actions such as gene transcription, protein stability, nucleo-cytoplasmic trafficking, cell cycle regulation, and apoptosis. However, its role in the pathobiology of biliary diseases, particularly in PLD, is unknown.

Protein SUMOylation involves the covalent and reversible binding of a small ubiquitin-like modifier (SUMO) to a substrate acceptor lysine residue. SUMO binding to proteins is regulated through an enzymatic cascade that involves the heterodimer E1 activating enzyme (SAE1/UBA2), E2 conjugating enzyme (UBC9) and an E3 ligating enzyme. Three SUMO paralogs (SUMO1–3) have been identified, SUMO1 being the most ubiquitous conjugated form.^5^ SUM02 and SUM03 share a sequence identity of 97%, and are therefore often referred as SUM02/3. Dysregulated protein SUMOylation has recently been demonstrated to contribute to human pathobiology, including liver conditions such as hepatocellular carcinoma (HCC) and non-alcoholic fatty liver disease (NAFLD).^6–8^ Prominently, S-adenosylmethionine (SAMe), mainly synthesized in the liver in the first reaction of the methionine cycle by the action of the enzyme methionine adenosyltransferase (MAT) I/III, is a natural inhibitor of UBC9, the sole E2-conjugating enzyme.^6^ The present study aims to unveil the role of SUMOylation in PLD and evaluate the therapeutic efficacy of SAMe.

## Material and methods

### Human samples

Cystic wall tissue from patients with PLD (n = 16), as well as healthy human gallbladder (n = 14) and liver (n = 14) biopsies were obtained from Radboud University Medical Center (Nijmegen, The Netherlands) and Donostia University Hospital (San Sebastian, Spain), respectively. Immunohistochemistry (IHC) was performed in paraffin-embedded liver tissue from the Mayo Clinic (Rochester, MN, USA), Radboud University Medical Center and Donostia University Hospital. Table SI summarizes the main demographic and clinical features of the patients included in the study. Research protocols were approved by the *Clinical Research Ethics Committees* of supporting Institutions [MSA-MMR-2017–01 (Donostia), 2012/317 (Radboud) and Mayo Clinic-IRB], and all patients signed written consent for the use of their samples for biomedical research.

### PLD animal model

The polycystic (PCK) rat (PCK/CrljCrl-*Pkhd1*^pck^/Crl; Charles River Laboratory) carries a spontaneous mutation in the polycystic kidney and hepatic disease 1 (*Pkhd1*) orthologous gene, responsible for an ARPKD/congenital hepatic fibrosis phenotype characterized by bile duct dilation and progressive development of intrahepatic biliary cysts (*i.e.,* isolated cysts at 6-months-old) and hepatic fibrosis.^9–10^ This animal model is widely used to study PLD pathobiology, although it has some differences with ADPLD/ADPKD phenotypes in terms of pathogenesis and fibrosis.^2^ 8-week-old PCK rats (n = 12) were treated with 20 mg/kg/day S-adenosyl-1-methionine disulfate p-toluenesulfonate (SAMe, kindly provided by Gnosis S.p.A., Desio, Italy) for 5 months by oral gavage. SAMe dosage was based on published studies.^11^ As non-treated controls, 8-week-old wild-type (WT, Sprague-Dawley, Charles River Laboratory) (n = 8) and PCK (n = 14) rats were maintained in parallel (Fig. S1). Analysis of serum biochemical markers, physical parameters, and endpoints of hepatic cystogenesis and fibrosis are described in the Supplementary data. All animal experimental procedures were approved by the *Animal Experimentation Ethics Committee* of Biodonostia Health Research Institute (CEEA17/007).

### Human and rat cholangiocyte primary cultures

Normal and polycystic human cholangiocytes, NHCs and PHCs respectively, were isolated as previously described.^12^ PHCs harbor a missense mutation [c.2515C>T, p.(Arg839Trp)] in the *GANAB* gene.^13^ Likewise, normal rat cholangiocytes (NRCs) and *Pkhd1-*mut rat cholangiocytes (PCKs) were isolated and cultured.^14^ All primary cells were cultured in fully supplemented DMEM/F-12 medium as previously described.^12^

### RNA isolation, retrotranscription and gene expression

RNA isolation was performed in human and rat liver tissue, as well as in cell cultures, using TRI Reagent (Sigma). Subsequently, reverse transcription and quantitative real-time PCRs (qPCRs) were carried out as described in the Supplementary data. Expression of glyceraldehyde-3-phosphate dehydrogenase (*GAPDH*) served as normalizing control. Primer sequences (Sigma) are included in Table S2 and the supplementary CTAT table.

### Immunohistochemistry

Detection of SUMO1 protein expression (*i.e.,* free SUMO1 and SUMO1-conjugated proteins), ubiquitin and p62 by IHC was carried out on paraffin-embedded sections from normal, ADPKD and ARPKD human liver tissues, as well as in normal rat and PCK tissues, as previously described.^12^ In addition, UBC9 protein expression was stained in normal and PCK rat liver tissues. The antibodies used are listed in Table S3 and the CTAT table.

### Immunoblotting

Changes in levels of SUMO1, SUMO1-conjugated proteins, ubiquitin-conjugated proteins, p62 and acetylated α-tubulin were analyzed by immunoblotting using whole cell lysates or SUMO1-immunoprecipitation (IP) cell lysates of cultured human cholangiocytes, as described in the Supplementary data.

### 3D cystic growth

Biliary cysts were isolated from PCK rats and cultured as previously reported.^12^ Twenty-four hours after isolation, 1.0 mM SAMe was added daily into the culture medium and the 3D expansion of cysts was monitored (at 24 h and 48 h) by light microscopy. The circumferential area of the cystic cholangioids was assessed using Image! software (National Institutes of Health, USA).

### Cell viability, proliferation and apoptosis

Cell viability, proliferation and apoptosis assays in human and rat cholangiocytes in culture were evaluated in the presence or absence of different compounds (i.e., SAMe and/or the proteasome inhibitor MG132 [Sigma]) as described in the supplementary information.

### Primary cilia

The number of ciliated PHCs in the presence or absence of SAMe in the culture media was analyzed by immunofluorescence, as described in the supplementary information.

### Cell transfection with short-hairpin RNA

PHCs were either transfected with control short-hairpin RNA (shRNA, Sigma) or shRNA against *UBE2I* (Sigma) using FuGENE HD (Promega), or left untransfected as experimental control. Stable transfected cells were obtained by selecting puromycin (Sigma) resistant cells, and *UBE2I* knockdown was verified by protein expression using immunoblotting. Details are outlined in the supplementary information.

### Immunoprecipitation

Whole cell lysates from NHCs and PHCs were incubated with Dynabeads Protein G (Invitrogen), which were crosslinked to SUMO1 or immunoglobulin G (IgG) antibodies (Abeam). The proteins were eluted from the beads with 2% SDS as described in the supplementary information.

### Mass spectrometry and proteomic analysis

Comparative shotgun proteomic analyses of control-shRNA and shRNA-[/BE2/samples, as well as SUMO1-IP lysates, were performed as described in the supplementary information.

### Proteasome activity

Proteasomal degradation of proteins was determined using the Proteasome-Glo™ Cell-Based Assay (Promega, Madison, WI) in NHCs and PHCs at baseline conditions and after incubation with 1.0 mM SAMe, following manufacturer’s instructions and as detailed in the supplementary information.

### Statistical analysis

Statistical analysis was performed using GraphPad Prism software (Version 8.3, San Diego, CA, USA). First, Shapiro-Wilk normality test was evaluated, and then, parametric Student’s *t* tests or non-parametric Mann-Whitney tests were applied to compare statistical significance between 2 groups. For comparisons between more than 2 groups, parametric one-way analysis of variance (ANOVA) or non-parametric Kruskal-Wallis tests followed by *a posteriori* Tukey or Dunns tests were used, respectively. Data are expressed as means ± SEM, and differences of *p* <0.05 were considered statistically significant.

For further details regarding the materials and methods used, please refer to the CTAT table and supplementary information.

## Results

### The SUMOylation machinery is upregulated in PLD

**Fig. 1.**
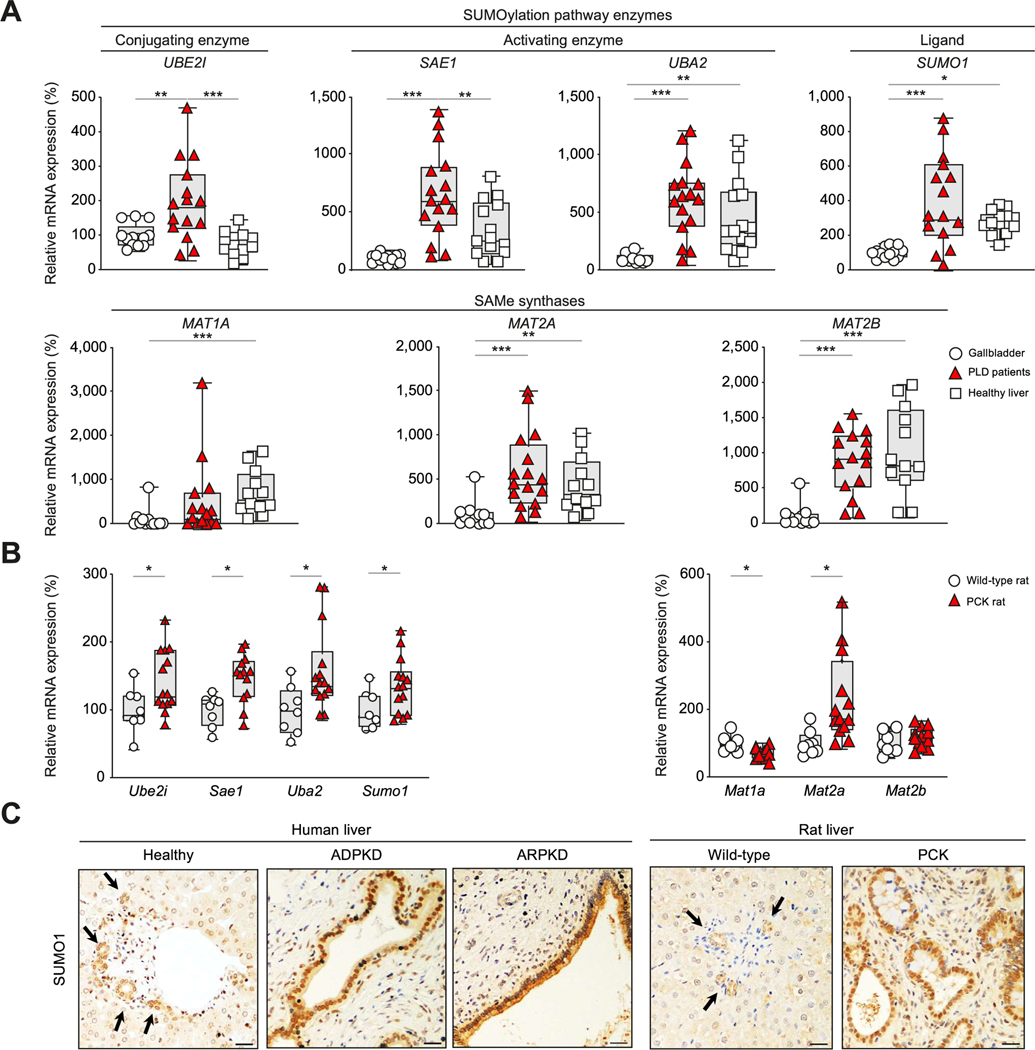
Expression levels of the SUMOylation and SAMe synthesis pathway components in cystic human and rat tissue. (A) mRNA expression of genes involved in SUMOylation and SAMe synthesis in healthy [human gallbladder (GB) (n = 14) and liver tissue (n = 14)] and cystic tissue of patients with PLD (n = 16), as well as (B) in liver tissue of WT (n = 8) and PCK (n = 14) rats. (C) Representative IHC images of SUMO1 and SUMO1-conjugated proteins in human and rat liver tissue. Scale bars: 500 μm. *p<0.05; **p<0.01; ***p <0.001 (one-way ANOVA tests, Kruskal-Wallis, Mann-Whitney or 2-tailed *t* tests). IHC, immunohistochemistry; PCK, polycystic; PLD, polycystic liver disease; SAMe, S-adenosylmethionine; WT, wild-type.

To determine whether SUMOylation and genes involved in SAMe synthesis are altered in PLD, expression (mRNA) levels were determined in cystic tissue of patients with PLD and compared to healthy human gallbladder (GB, which is enriched in cholangiocytes) and liver tissue. Cystic tissue of patients with PLD showed higher expression of E2 conjugating enzyme, UBC9 (*UBE2I*), and E1 activating enzyme subunit 1, SAE1 (*SAE1*), when compared to both GB and liver tissues (Fig. 1A). In addition, E1 activating enzyme subunit 2, UBA2 (*UBA2*), and the substrate itself, SUMO1 (*SUMO1),* were overexpressed in cystic tissue compared to GB tissue (Fig. 1A). On the other hand, the genes involved in the generation of MAT, which catalyze SAMe synthesis, *MAT2A* and *MAT2B* (widely expressed in extrahepatic tissues), were found overexpressed in PLD when compared to GB tissue (Fig. 1A). No significant differences were seen in *MATIA* (mainly expressed in liver tissue) or *MAT2A-B* expression between PLD and liver tissue, whereas decreased *MATIA* expression was observed in GB compared to liver tissue (Fig. 1A). In parallel, the expression (mRNA) levels of the key enzymes involved in SUMOylation and SAMe synthesis were measured in liver tissue of normal and PCK rats. Similar trends of expression were seen in liver tissue of PCK compared to WT rats, with upregulation of *Ube2i, Sae1, Uba2, Sumo1* and *Mat2a.* On the other hand, the expression of *Mat1a* was found downregulated, whereas *Mat2b* expression remained unchanged, in PCK vs. WT livers (Fig. 1B). Moreover, immunostaining of the liver showed an enrichment of SUMO1 (*i.e.,* combination of free SUMO1 ligand and SUMO1-conjugated proteins) in the cystic epithelium of patients with ADPKD and ARPKD, as well as in PCK rats, compared to the biliary epithelium of healthy controls (Fig. 1C). In particular, SUMO1 overexpression was preferentially found in the nucleus of cystic cholangiocytes, indicating an upregulation of SUMO1 conjugation to proteins.

**Fig. 2.**
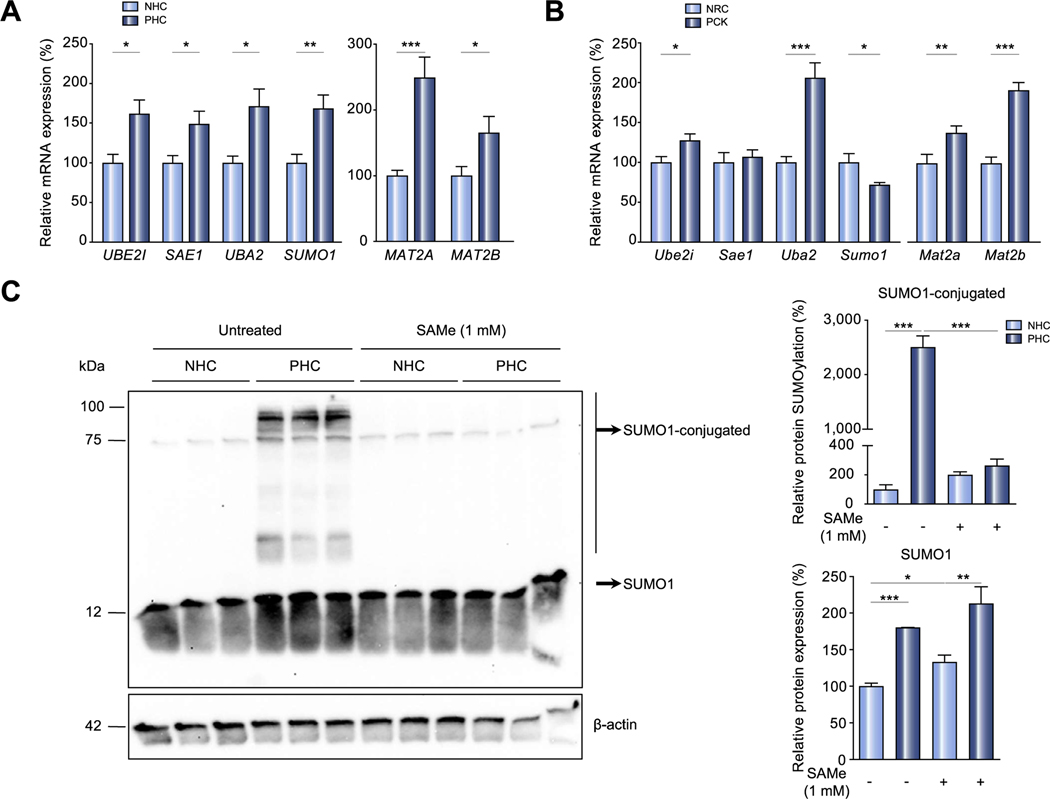
Expression levels of the SUMOylation and SAMe synthesis pathway components in human and rat cystic cholangiocytes *in vitro*. (A) mRNA expression of genes involved in SUMOylation and SAMe synthesis in NHCs (n = 6) and PHCs (n = 6), as well as (B) in NRCs (n = 11) and PCK cholangiocytes (n = 11). (C) Representative immunoblot and quantification of SUMO1 and SUMO1-conjugated proteins in NHCs and PHCs, in the presence or absence of SAMe (n = 3). *p <0.05; **p <0.01; ***p <0.001 (one-way ANOVA tests, Kruskal-Wallis, Mann-Whitney or 2-tailed *t* tests). NHCs, normal human cholangiocytes; NRCs, normal rat cholangiocytes; PHCs, polycystic human cholangiocytes; PCKs, polycystic rat cholangiocytes; SAMe, S-adenosylmethionine.

To examine whether SUMOylation and SAMe synthesis were also dysregulated at the cellular level, the same array of genes was evaluated in primary cultures of NHCs, PHCs, NRCs and PCK cholangiocytes. In human cells, upregulation of every gene was observed in PHCs (Fig. 2A), corroborating the results obtained in tissue. Comparable alterations were observed in PCK cholangiocytes, with the exception of *Sae1* and *Sumo1.* While the expression of *Sae1* is comparable in both normal and cystic rat cholangiocytes, *Sumo1* is downregulated in PCK cholangiocytes compared to NRCs (Fig. 2B). On the other hand, *MATIA* or *Matla* expression was not detected in either human or rat cell culture (data not shown). Importantly, the analysis of SUMOylation at the protein level demonstrated substantially higher levels of SUMO1 and SUMO1-conjugated proteins in PHCs compared to NHCs (Fig. 2C). In view of the hyper-SUMOylation state in cystic cholangiocytes, the effect of SAMe administration, a natural inhibitor of UBC9, was further evaluated.^6^ Incubation of PHC with SAMe abolished the increased levels of SUMO1-conjugated proteins in PHCs, restoring the SUMOylation status to similar levels seen in NHCs (Fig. 2C). On the other hand, the levels of free SUMO1 were unaffected by SAMe in PHC (Fig. 2C).

### SAMe halts hepatic cystogenesis and fibrosis in PCK rats

**Fig. 3.**
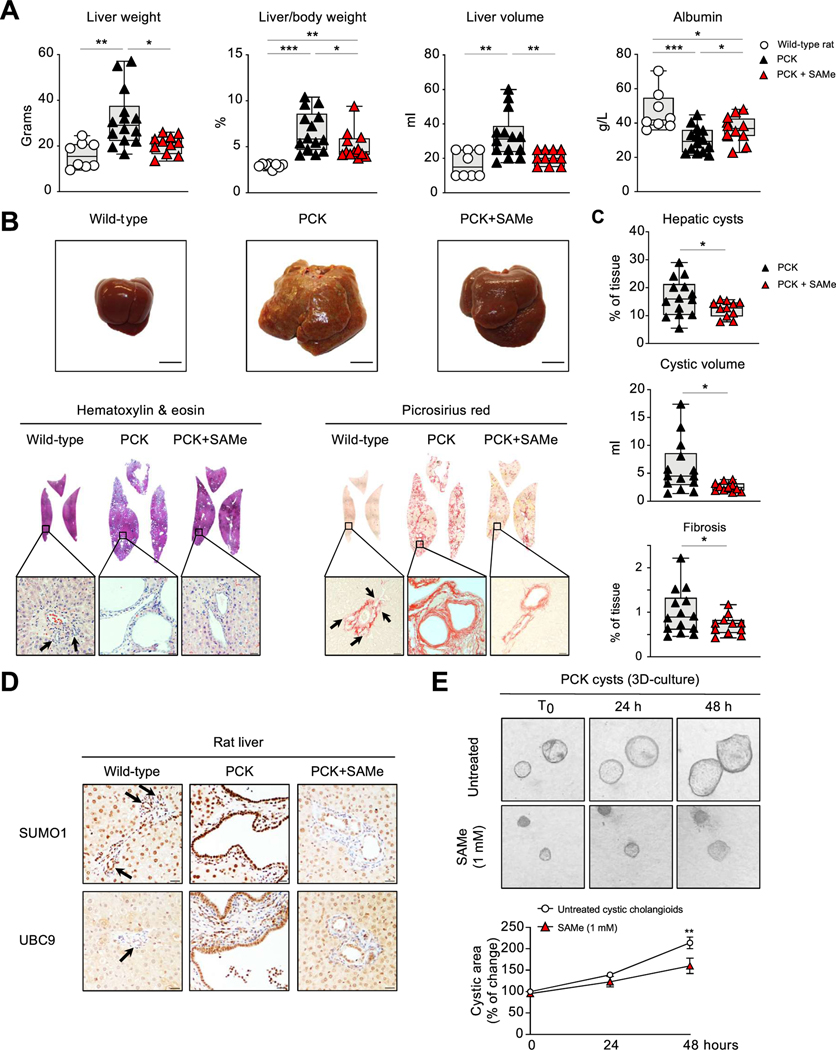
Effect of SAMe administration on hepatic cystogenesis and fibrosis in PCK rats. (A) Physical parameters and serum albumin levels in WT (n = 8) and PCK (untreated [n = 14] and SAMe-treated [n = 12]) rats. (B) Representative liver images (scale bar: 1 cm), H&E and picrosirius red staining (scale bar: 500 μm). (C) Hepatic cystogenesis and fibrosis quantification in PCK (untreated and SAMe-treated) rats. (D) Representative IHC images of SUMO1 and UBC9 in rat liver tissue. (E) 3D-cultured PCK cysts in the presence/absence of SAMe (n = 12) (T_0_: time zero). *p <0.05; **p <0.01; ***p <0.001 (one-way ANOVA, Kruskal-Wallis, Mann-Whitney or 2-tailed *t*-tests, except fibrosis [one-tailed *t* test]). IHC, immunohistochemistry; PCK, polycystic: SAMe, S-adenosylmethionine; WT, wild-type.

To further investigate whether modulation of SUMOylation with SAMe represents a potential therapeutic strategy for patients with PLD, PCK rats were chronically administered with SAMe (20 mg/kg/day for 5 months). Administration of SAMe halted hepatic cystogenesis in PCK rats, reflected by the lower liver weight, liver/body weight ratio and liver volume, when compared to non-treated PCK rats (Fig. 3A). In addition, a partial normalization of the decreased serum albumin levels of PCK rats was evident after SAMe treatment (Fig. 3A). Mitigation of hepatic cystogenesis was further strengthened by changes seen at macroscopic and microscopic levels (Fig. 3B). Hepatic cystic and fibrotic areas, as well as liver cystic volume from PCK rats, were reduced after SAMe treatment (Fig. 3C). Importantly, SUMO1 (*i.e*., combination of free SUMO1 ligand and SUMO1-conjugated proteins) and UBC9 protein overexpression in PCK biliary cysts was lowered after SAMe treatment (Fig. 3D). On the other hand, SAMe treatment did not alter: bile flow; the levels of serum biomarkers of liver injury (*i.e.*, alanine aminotransferase and aspartate aminotransferase [AST]) and cholestasis (*i.e.*, alkaline phosphatase); the expression of tissue biomarkers of liver injury (*i.e.* pro-inflammatory and pro-fibrotic genes) and SUMOylation genes; or renal cystogenesis (Fig. S2; Table S4). Finally, 3D cultures of biliary cysts isolated from PCK rats were incubated in the presence or absence of SAMe (1.0 mM) and followed up for 3 days. In these conditions, PCK cystic cholangioids grew overtime (Fig. 3E). Interestingly, the presence of SAMe (for 48 h) decreased the cystic cholangioid growth compared to control conditions (Fig. 3E), resembling the effects observed with SAMe *in vivo.*

### SAMe inhibits cystic growth *in vitro* by impacting on cell survival, proliferation and differentiation

**Fig. 4.**
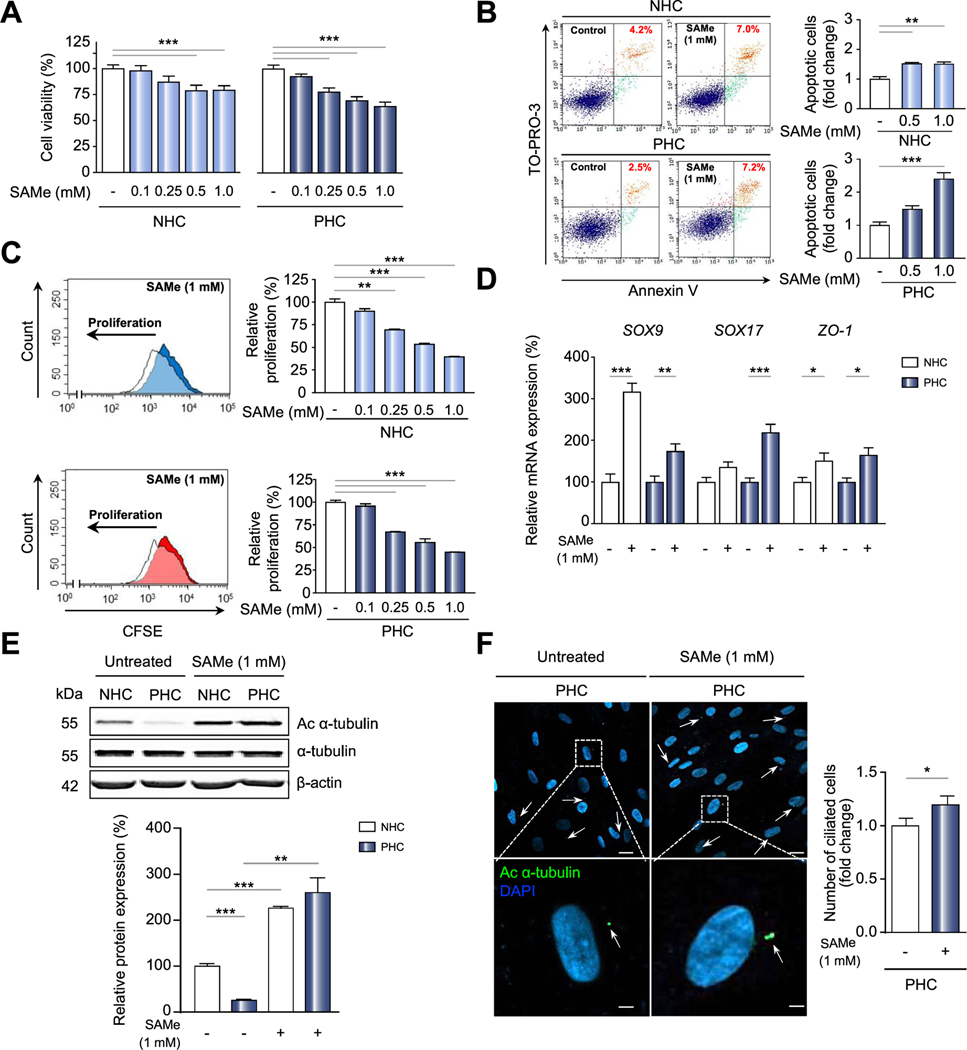
Biological impact of SAMe on survival and proliferation of normal and cystic cholangiocytes in culture. Cell (A) viability (n = 6), (B) apoptosis (n = 4) and (C) proliferation (n = 3) in untreated or SAMe-treated NHCs and PHCs. (D) mRNA levels of epithelial and polarity markers in the presence/absence of SAMe. (E) Immunoblot and quantification of acetylated oi-tubulin in cultured PHCs, in the presence/absence of SAMe (n = 6). (F) Immunofluorescence images of acetylated α-tubulin in PHCs (primary dlium in green and nucleus in blue) in the presence/absence of SAMe (n = 24) (scale bars: 20 μm, 4 μm [cropped]). *p <0.05; **p <0.01; ***p <0.001 (one-way ANOVA Kruskal-Wallis, Mann-Whitney or 2-tailed *t*-tests). NHCs, normal human cholangiocytes; PHCs, polycystic human cholangiocytes; SAMe, S-adenosylmethionine.

To further address the direct effects of SAMe on cystic cholangiocytes, cell survival, proliferation and apoptosis assays were performed. First, cell viability was measured using different concentrations of SAMe in both human and rat normal and cystic cholangiocyte cultures. Of note, SAMe decreased cell viability of both PHCs and PCK cholangiocytes in a dose-dependent manner when compared to their respective untreated controls (Fig. 4A and Fig. S3A, respectively); moreover, the effect of SAMe on cell viability was significantly stronger in cystic cholangiocytes than in normal cholangiocytes (both human and rat). Since cell viability is the result of the balance between survival and proliferation, both cellular processes were investigated next. The exposure of cystic cholangiocytes to SAMe induced a dose-dependent apoptosis in both PHCs and PCK cholangiocytes compared to normal controls where SAMe had no or minor effect (Fig. 4B and Fig. S3B, respectively). A main feature of cystic cholangiocytes is their baseline hyperproliferation compared to normal cholangiocytes.^2,14^ Interestingly, SAMe caused a dose-dependent reduction of cell proliferation in both PHC and PCK cholangiocytes (Fig. 4C and Fig. S3C, respectively), which was of greater magnitude in the latter. Furthermore, incubation of PHCs and NHCs with 1.0 mM SAMe induced the expression of biliary differentiation (*SOX9* and *SOX17*) and cell polarity (ZO-J) markers (Fig. 4D). Notably, SAMe increased the levels of acetylated α-tubulin (a primary cilium marker) in PHCs, as well as the number of ciliated cells (Figs. 4E,F, respectively).

### Experimental knockdown of *UBE2I* mimics SAMe effects in cystic cholangiocytes *in vitro*

**Fig. 5.**
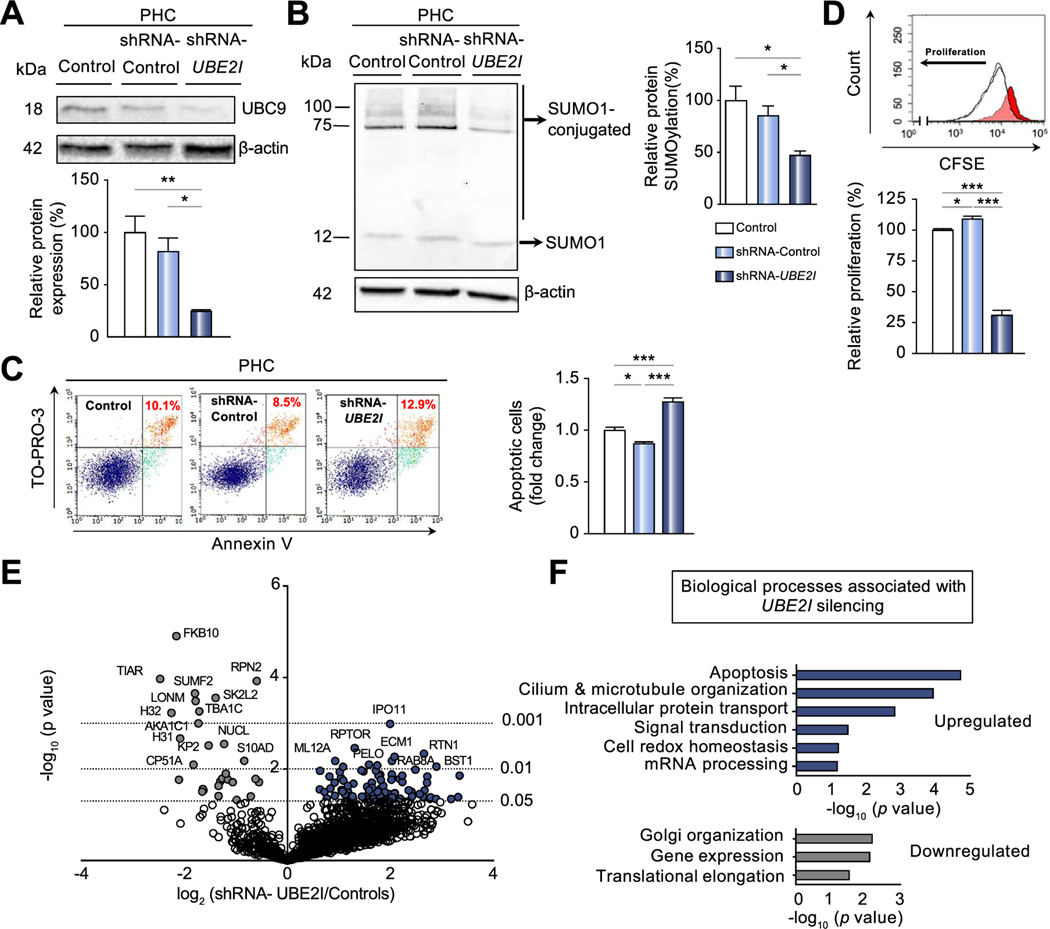
Biological effects of *UBE2I* experimental knockdown in PHCs *in vitro*. (A) Representative immunoblot and quantification of UBC9 and (B) SUMOl and SUMOl-conjugated proteins in shRNA-l/BE27 and control (non-transfected and shRNA-control) PHC in culture. (C) Apoptosis (n = 4) and (D) proliferation in preceding groups (n = 3). (E) Volcano plot of proteins quantified and considered in differential analysis (n = 2,109) by mass spectrometry comparing fold enrichment in shRNA-l/B£2/to controls. Proteomic analyses of the significant dysregulated proteins (n = 120) in the aforementioned groups by (F) gene ontology. *p <0.05; **p <0.01; ***p <0.001 (one-way ANOVA or Kruskal-Wallis tests). PHCs, polycystic human cholangiocytes; shRNA, short-hairpin RNA.

As mentioned above, SAMe has been described to regulate SUMOylation by inhibiting UBC9, the sole E2-conjugating enzyme in SUMOylation. To validate whether the functional effects exerted by SAMe were through UBC9 inhibition, stable shRNA-mediated *UBE2I* knockdown in PHCs was evaluated. Immunoblotting confirmed *UBE2I* silencing, as UBC9 protein levels were reduced by 65–75% in shRNA-*UBE2I* compared shRNA-control or non-transfected cystic cholangiocytes respectively (Fig. 5A). In accordance, shRNA-*UBE2I* PHCs presented reduced protein SUMOylation (Fig. 5B). Consistent with SAMe treatment, UBC9 depletion had pro-apoptotic and antiproliferative consequences in PHCs compared to both controls (Figs. 5C,D). Furthermore, shotgun proteomic analyses were performed to determine the protein profile regulated by UBC9 in cystic cholangiocytes and their potential role in the pathogenesis of PLD. In total, 3,681 and 3,585 proteins were identified in controls (non-transfected cystic cholangiocytes and shRNA-control) and shRNA-*UBE2I* transfected PHCs, respectively (Fig. S4A). Among the differentially expressed proteins, 29 proteins (24%) were downregulated (grey) and 91 proteins (76%) upregulated (dark blue) in shRNA-*UBE2I* transfected PHCs compared to controls (Figs. 5E; Fig. S4B; Table S5). Gene ontology (GO) analysis indicated that the upregulated proteins in shRNA-*UBE2I* transfected PHCs are involved in different cellular processes including apoptosis (*i.e.*, TRADD, PEA15, PDPK1, PML), ciliogenesis (*i.e.*, DYNLL2, RAB8, TAGLN2, EHD1 ), as well as intracellular transport of proteins (*i.e.*, IPO11, RABGAP1) (Fig. 5F). Finally, the STRING analysis showed numerous protein-protein interactions (PPIs) between the identified proteins, with a PPI enrichment *p* value of 2.95E-06, indicating that the predicted functional associations between these proteins are significant (Fig. S4C).

### Most SUMOylated proteins that are elevated in cystic cholangiocytes are involved in protein biogenesis

**Fig. 6.**
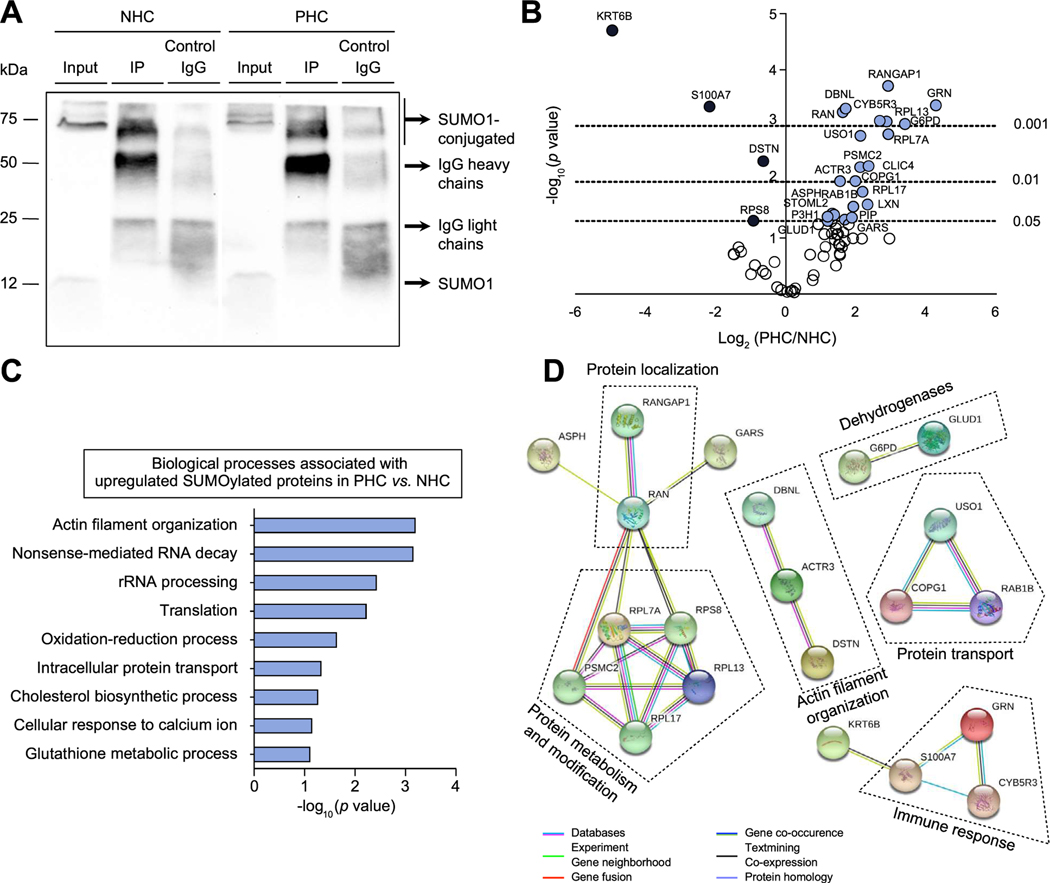
Comparative SUMOylated protein profile between PHCs and NHCs in culture after SUMOl-immunoprecipitation. (A) Representative immunoblot of SUMO-IP in NHC and PHC. (B) Volcano plot of all identified SUMOl-IP proteins (n = 67) by mass spectrometry comparing fold enrichment in PHCs to NHCs. Proteomic analyses of significant identified proteins (n = 26) in aforementioned groups by (C) gene ontology and (D) PPI network. Line color in PPI network indicates type of interaction evidence. IP, immunoprecipitation; NHCs, normal human cholangiocytes; PHCs, polycystic human cholangiocytes; PPI, protein-protein interaction.

To elucidate and compare the SUMOylated proteins in both NHCs and PHCs, SUMO1-conjugated proteins were isolated by IP and identified by mass spectrometry (MS). Immunoblotting confirmed enrichment of SUMO1-IP proteins compared to inputs in respective cell cultures (Fig. 6A). Next, shotgun proteomic analyses identified 67 SUMOylated proteins in NHCs and/or PHCs. Among them, 26 proteins were differentially expressed between both cell lines, with increased levels of 22 proteins (85%) and decreased levels of 4 proteins (15%) in PHCs compared to NHCs (Fig. 6B; Fig. S5; Table S6). GO analysis pointed out that most of the SUMOylated proteins found increased in PHCs participate in biological processes related to actin filament organization (*i.e*. ACTR3, RAN, DBNL), oxidation-reduction process (GLUD1, ASPH, G6PD), and the protein translation machinery (*i.e*. RPL7A, RPL17) (Fig. 6C). The PPI analysis identified different associations predominantly linked to the protein translational machinery (*i.e.* protein modification, transport, localization and metabolism) (Fig. 6D). Interestingly, PSMC2, a regulatory subunit of the 26S proteasome, was found SUMOylated and upregulated in PHCs, suggesting that the modulation of the proteasome activity might be a potential mechanism behind the therapeutic effects of SAMe.

### SAMe reduces the proteasome activity in cystic cholangiocytes and induces stress-related apoptosis

**Fig. 7.**
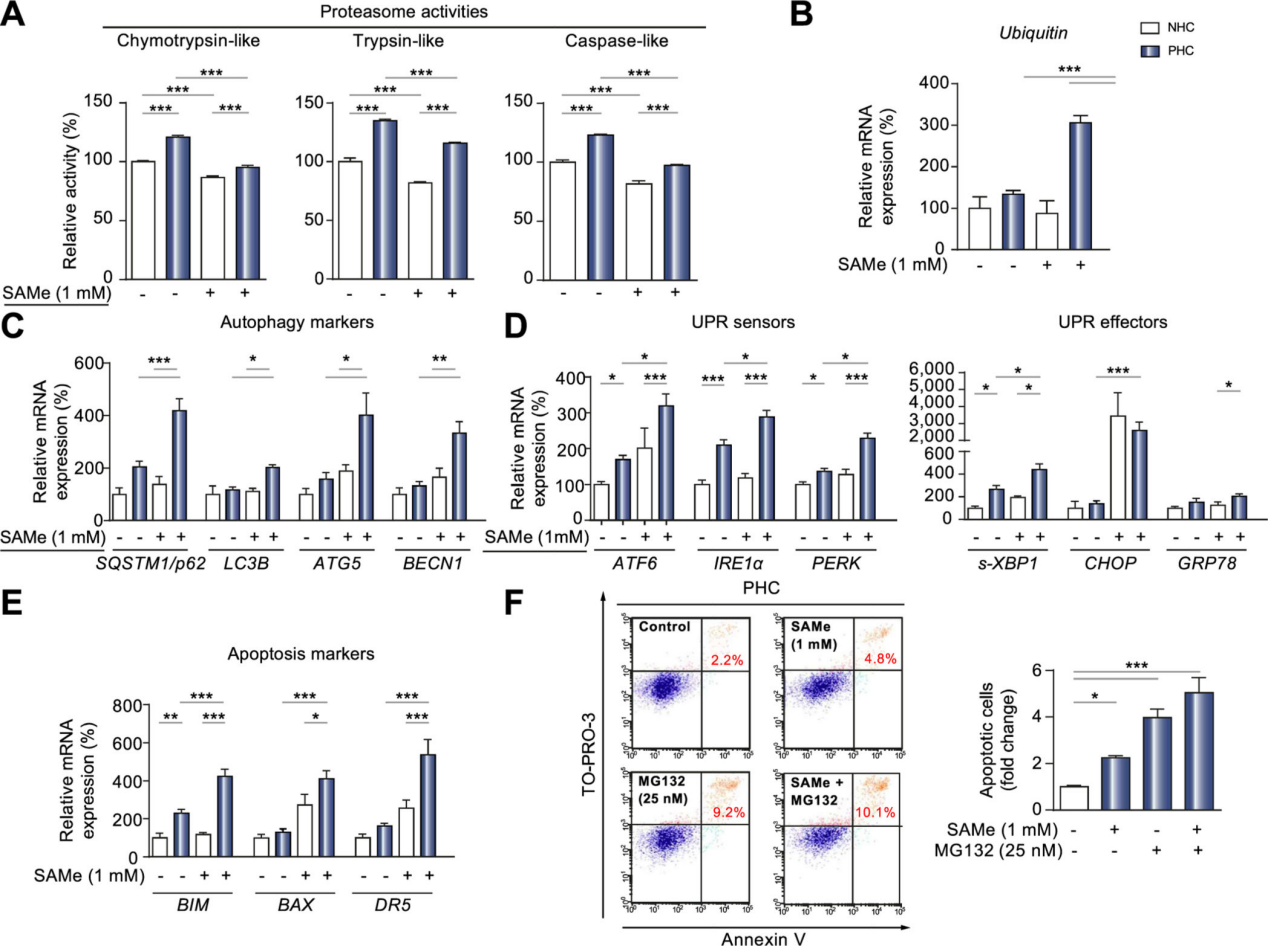
SAMe reduces proteasome activity in cystic cholangiocytes and induces stress-related apoptosis *in vitro*. (A) Relative proteasome activity of NHCs and PHCs under baseline conditions or SAMe treatment (n = 4). mRNA levels of (B) ubiquitin (C) autophagy markers (D) UPR sensors and effectors, and (E) apoptosis markers in the presence/absence of SAMe. (F) Apoptosis (n = 3) in untreated, SAMe-treated, MG132-treated, and combination of SAMe and MG132-treated PHCs. *p <0.05; **p <0.01; ***p <0.001 (one-way ANOVA or Kruskal-Wallis tests). NHCs, normal human cholangiocytes; PHCs, polycystic human cholangiocytes; SAMe, S-adenosylmethionine; UPR, unfolded protein response.

Based on the identification of increased PSMC2 SUMOylation levels in PHCs and the previously described proteasome hyperactivity of PHCs vs. NHCs^4^ - together with the baseline increase of ubiquitinated proteins and p62 (marker of autophagy) expression in cystic tissue from PLD patients and PCK rats compared to controls (Fig. S6A–D) - we evaluated the impact of SAMe on proteasome activity. Interestingly, the chymotrypsin-, trypsin- and caspase-like proteasome hyperactivities of PHCs were normalized to baseline NHC values by SAMe administration (Fig. 7A). Consequently, SAMe induced transcriptional activation of ubiquitin expression in cystic cholangiocytes (Fig. 7B). Nonetheless, this effect was not mirrored by an accumulation of ubiquitinated proteins in PHCs after SAMe treatment (Fig. S6C,D). In this regard, several autophagy markers (*i.e*., *SQSTM1/p62, LC3B, ATG5* and *Beclin-1*) were overexpressed in cystic cholangiocytes after SAMe treatment (Fig. 7C), suggesting that autophagy activation might compensate, at least in part, for the loss of proteasome degrading capacity. Interestingly, accumulation of p62 and ubiquitin-conjugated proteins was found in the liver of SAMe-treated PCK rats (Fig. S6E,F), suggesting the inability of autophagy to fully compensate for the long-term accumulative effects of SAMe-driven proteasome inhibition. Next, the effect of SAMe on the unfolded protein response (UPR) was assessed; we observed increased expression of the UPR sensors (*i.e.*, *ATF6, IRE1α* and *PERK*) and effectors (*i.e.*, *s-XBP1, CHOP* and *CRP78*) on SAMe-treated cystic cholangiocytes (Fig. 7D). Furthermore, various stress-related apoptosis markers (*i.e.*, *BIM, BAX* and *DR5*) were likewise elevated in PHCs upon SAMe administration (Fig. 7F). Notably, the combination of SAMe and MG132, a selective and strong 26S proteasome complex inhibitor, did not have additive or synergic effect on the apoptosis of PHCs (Fig. 7G), suggesting a common pathway of apoptosis induction for these 2 compounds.

## Discussion

Our data showed that the expression levels of nearly all core SUMO pathway components are upregulated in both hepatic cystic tissue and cultured cystic cholangiocytes from humans and mutant rats with PLD, compared to (corresponding normal) controls. The relevance of our findings is corroborated by the presence of increased levels of SUMO1-conjugated proteins in cystic tissue and cholangiocytes that promote hepatic cystogenesis *in vitro* and *in vivo.* SUMOylation may be substantially enhanced in response to diverse stimuli, as illustrated in various conditions such as cancer, certain neurological conditions, heart failure and diabetes mellitus.^5^ Earlier reports observed upregulation of SUMO1 and UBC9 in colorectal cancer and HCC, whereas increased expression of SAE1/UBA2 was found in HCC.^15^ Moreover, increased SUMOylation of proteins has been associated with the ER stress-driven UPR, as it directs the cell into survival or death depending on the degree of stress.^16^ Indeed, as a result of the mutated genes in PLD, misfolded proteins accumulate in the ER, thereby inducing prolonged ER stress and subsequently UPR hyperactivity.^4^ This suggests that enhanced protein SUMOylation in PLD could be related with the UPR, driving disease progression.

Considering the inhibitory capacity of SAMe on the SUMOylation enzyme UBC9, the homeostasis of this essential endogenous metabolite was analyzed in detail under baseline conditions in PLD. SAMe plays a pivotal role in transmethylation, transsulfuration and polyamine biosynthesis, thus impaired SAMe homeostasis may lead to pathological conditions. SAMe is synthesized from methionine by the enzymes MATI and MATIII (gene products of *MAT1 A),* which are primarily expressed in the liver, and MATH (gene product of *MAT2A* and *MAT2B*), which is widely expressed in extrahepatic tissues.^17^ Data derived from clinical studies support the favorable tolerability profile of SAMe supplementation, also improving the serum biochemistries (i.e., AST and bilirubin) in certain chronic liver conditions.^18^ The most common side effect of SAMe is nausea and, less frequently, diarrhea, abdominal discomfort, or vomiting.^19^ In view of the excellent safety profile of SAMe, it is likely that prolonged treatment for a condition such as PLD will be acceptable. Dysregulated SAMe homeostasis was observed in PLD, with increased *MAT2A* and *MAT2B* expression (and absence of expression of *MAT1A*) in both human and rat cystic cholangiocytes compared to normal cholangiocytes. Notably, the administration of SAMe normalized the protein hyper-SUMOylation levels and upregulated several markers of biliary and epithelial differentiation in cystic cholangiocytes *in vitro.* Therefore, in order to study the impact of the protein hyper-SUMOylation state in PLD, SAMe was administrated to experimental models of PLD *in vivo* and *in vitro.* Indeed, chronic treatment of PCK rats with SAMe (20 mg/kg/day for 5 months) mitigated hepatic cystogenesis and fibrosis compared to untreated PCK controls. Importantly, liver weight, liver/body weight ratio and liver volume of treated PCK rats were reduced, coupled with improved serum albumin levels. These therapeutic benefits mediated by SAMe were associated with the normalization of SUMO1 and UBC9 protein levels in cystic cholangiocytes (in both nucleus and cytoplasm), and with pro-apoptotic and antiproliferative responses. However, the possibility that other molecular processes modulated by SAMe are involved in the observed effects cannot be excluded. Interestingly, acute treatment with higher doses of SAMe *in vivo* (50 or 100 mg/kg/day for 2 months) barely led to changes in physical and serum biochemical parameters, apart from improved blood urea values (in the 50 mg/kg/day SAMe group) and improved AST values (in the 100 mg/kg/day SAMe group) (Fig. S7; Table S7). This implies that SAMe might be more efficient as a long-term rather than short-term treatment in PLD. Moreover, oral SAMe treatment was well tolerated and no adverse effects were noticed in all of the treated PCK groups (*i.e.,* 20 mg, 50 mg and 100 mg SAMe), corresponding with published data on SAMe clinical trials.^18^

UBC9 has a critical role as the single E2 conjugating enzyme in the multistep pathway of SUMOylation. Since a high proportion of the SUMO substrates appear to be nuclear resident proteins in PLD, alterations in UBC9 expression and activity may impact numerous cellular processes related to gene transcription. To confirm whether the effects of SAMe are due to UBC9 inhibition, as well as to identify potential UBC9 targets by MS, stable shRNA-*UBE2I* transfected PHCs were investigated. Consistent with the results seen in SAMe-treated PHCs, *UBE2I* silencing achieved comparable pro-apoptotic and antiproliferative effects. Furthermore, proteomic analyses of *UBE2I* shRNA-transfected PHCs identified an extensive number (>100) of differentially expressed proteins, with the majority (~75%) being upregulated compared to controls. *UBE2I* silencing in PHCs elicited functional enrichments of proteins involved in biological processes such as protein transport, ciliogenesis, and apoptosis. To identify specific SUMO target proteins, proteomic analyses of immunoprecipitated SUMO1 proteins were performed. The number of SUMOylated proteins was increased in PHCs compared to NHCs. Several biological processes of these SUMOylated proteins were analogous to the ones found dysregulated under *UBE2I* silencing in PHC, in particular protein biogenesis, transport and actin filament organization. On another note, the abundance of a SUMOylated protein does not reveal whether its function is altered (i.e., reduced, increased or unchanged), yet it allows for identification of SUMO targets, such as PSMC2, a subunit of the 26S proteasome, in which the SUMOylation status could play an important role in protein stability and/or function. Interestingly, SAMe was able to normalize the baseline proteasome hyperactivity in cystic cholangiocytes; this resulted in transcriptional activation of ubiquitin, and induction of autophagy markers, UPR and stress-related apoptosis. Furthermore, combined administration of SAMe and MG132 did not increment the apoptotic effects exerted by MG132 alone, indicating a shared mechanism of apoptosis induction for these 2 compounds. This indicates that SUMOylation of PSMC2 may enhance proteasome stability, promoting the degradation of un/misfolded proteins, partially alleviating ER stress and leading to cell survival. Therefore, SAMe administration, and the consequent PSMC2 de-SUMOylation, could affect the 26S proteasome conformation and inhibit proteasomal degradation of proteins, ultimately resulting in increased apoptosis due to exacerbated stress, as occurs in the MG132-mediated proteasomal inhibition.^17,20^ Of note, increased apoptosis was also observed in *UBE2I*-silenced PHCs, which presented an upregulation of various apoptosis-related proteins (*i.e.,* TRADD, PEA15, PDPK1, PML). The chaperone CDC37 was found to be downregulated in shRNA-*UBE2I* cystic cholangiocytes; this could affect protein folding, further increase cellular stress and contribute to enhanced apoptosis. Importantly, duration and severity of ER stress can determine the switch between survival and apoptosis.^21^ Thus, SUMOylation inhibition may lead to apoptosis in highly stressed cells, whereas it may favor cell differentiation, polarization and primary cilium restoration in less-stressed PHCs, suggesting that fine tuning of stress is crucial for cell fate.

**Fig. 8.**
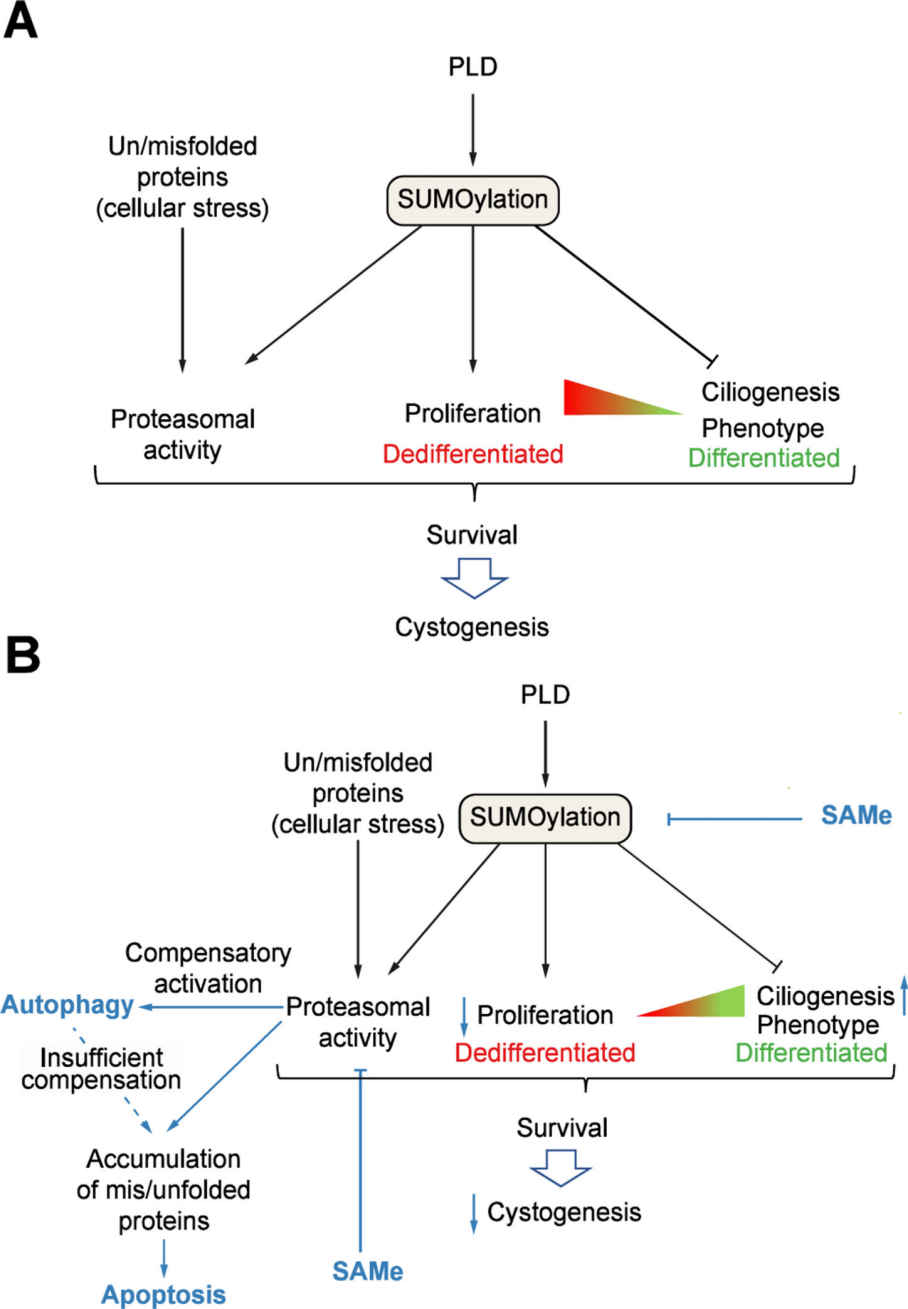
Working model. (A) Cystic cholangiocytes are characterized by altered protein dynamics, which promote the activation of adaptive PTM processes, including SUMOylation, resulting in the induction of pro-survival mechanisms and hepatic cystogenesis. (B) Targeting UBC9 with SAMe reduces protein SUMOylation and halts hepatic cystogenesis by increasing apoptosis, reducing proliferation and promoting differentiation of cystic cholangiocytes. PTM, post-translational modifications; SAMe, S-adenosylmethionine.

In summary (Fig. 8), this study reveals the important role of the SUMOylation of proteins in the hepatic cystogenesis of PLD. Cystic cholangiocytes are characterized by abnormal proteostasis associated with adaptive PTM mechanisms that promote protein dynamics. In particular, increased UBC9-dependent SUMOylation of certain proteins plays a central role in the pathogenesis of PLD, promoting the activation of pro-survival pathways in cystic cholangiocytes. Targeting of UBC9 with SAMe halts hepatic cystogenesis, thus representing a promising new therapeutic strategy for patients with PLD that deserves clinical evaluation.

## Supplementary Material

Suppl Material 2

Suppl Material 1

Suppl Material 3

## Highlights

- PTM abnormalities govern the pathogenesis of polycystic liver disease.
- Cystic cholangiocytes show increased UBC9-dependent protein SUMOylation.
- SUMOylation exerts adaptive protein dynamics and pro-survival mechanisms.
- Targeting of UBC9 with SAMe attenuates hepatic cystogenesis and fibrosis.
- SAMe arises as a promising therapeutic agent for patients with PLD.

## Abbreviations

ADPKD: autosomal dominant polycystic kidney disease
ADPLD: autosomal dominant polycystic liver disease
ALG8: alpha-1,3-glycosyltransferase
ALG9: alpha-1,2-mannosyltransferase
ARPKD: autosomal recessive polycystic kidney disease
AST: aspartate aminotransferase
ATF6: activating transcription factor 6
ATG5: autophagy related 5
BAX: Bcl-2 associated X apoptosis regulator
BECN1: Beclin-1
BIM: Bcl-2-like protein 11
CHOP: DNA damage-inducible transcript 3
DR5: death receptor 5
ER: endoplasmic reticulum
GANAB: glucosidase II alpha subunit
GAPDH: glyceraldehyde-3-phosphate dehydrogenase
GB: gallbladder
GO: gene ontology
HCC: hepatocellular carcinoma
GRP78: heat shock 70kD protein 5
IHC: immunohistochemistry
IgG: immunoglobulin G
IP: immunoprecipitation
IRE1α: serine/threonine-protein kinase/endoribonuclease inositol-requiring enzyme lα
LC3B: microtubule-associated protein 1 light chain 3 beta
MS: mass spectrometry
MAT: methionine adenosyltransferase
MATIA: methionine adenosyltransferase 1 A
MAT2A: methionine adenosyltransferase 2 A
MAT2B: methionine adenosyltransferase 2B
NAFLD: non-alcoholic fatty liver disease
NHC: normal human cholangiocytes
NRC: normal rat cholangiocytes
PCK: polycystic
PCK: polycystic rat cholangiocyte
PERK: PKR-like ER kinase
PHC: polycystic human cholangiocytes
PKD1: polycystic kidney disease 1
PKD2: polycystic kidney disease 2
PKHD1: polycystic kidney and hepatic disease 1
PLD: polycystic liver disease
PPI: protein-protein interactions
PRKCSH: protein kinase C substrate 80K-H
PSMC2: 26S protease regulatory subunit 7
PTM: post-translational modification
qPCR: real-time quantitative PCR
SAE1: SUMO1 activating enzyme subunit 1
SAE2: SUMO1 activating enzyme subunit 2
SAMe: S-adenosylmethionine
SEC61B: SEC61 translocon beta subunit
SEC63: SEC63 homolog protein translocation regulator
shRNA: short-hairpin RNA
SOX9: SRY-box 9
SOX17: SRY-box 17
SQSTM1: Sequestosome 1
SUMO: small ubiquitin-related modifier
s-XBPl: spliced X-box binding protein 1
UBA2: ubiquitin-like modifier activating enzyme 2
UBC9: SUMO-conjugating enzyme UBC9
UBE2I: ubiquitin conjugating enzyme E21
UPR: unfolded protein response
WT: wild-type
ZO-1: zona occludens-1
