# Supplementary material for: Targeting UBC9-mediated protein hyper-SUMOylation in cystic cholangiocytes halts polycystic liver disease in experimental models
Source: J Hepatol. Author manuscript; Available in PMC 2021 May 27. (PMC8157180; doi:10.1016/j.jhep.2020.09.010)
Supplement: Suppl Material 2 [file NIHMS1666894-supplement-Suppl_Material_2.pdf]

Journal of Hepatology

CTAT methods

Tables for a "Complete, Transparent, Accurate and Timely account" (CTAT) are now mandatory for all revised submissions. The aim is to enhance the reproducibility of methods.

- Only include the parts relevant to your study
- Refer to the CTAT in the main text as 'Supplementary CTAT Table'
- Do not add subheadings
- Add as many rows as needed to include all information
- Only include one item per row

If the CTAT form is not relevant to your study, please outline the reasons why:

1.1 Antibodies

Name	Citation	Supplier	Cat no.	Clone no.
Mouse monoclonal anti β-actin	Uribarri AD *et al*. Gut 2015.	Sigma	A5316	AC-74
Rabbit monoclonal anti-SUMO1	Yang, D *et al*. Blood 2003.	Abcam	ab32058	Y299
Rabbit monoclonal anti-SUMO1 (BSA and Azide free)	Not available	Abcam	ab219724	Y299
Rabbit monoclonal anti-UBC9	He, X *et al*. Nat Chem Biol 2017.	Abcam	ab75854	EP2938Y
Rabbit monoclonal anti-Ubiquitin	Zhang K *et al*. Nat Commun 2018.	Abcam	ab134953	EPR8830
Mouse monoclonal anti-p62		Abcam	ab56416	X
Mouse monoclonal anti-acetylated α-tubulin	Caballero-Camino FJ *et al*. Hepatology 2020.	Sigma-Aldrich	T6793	6-11 B-1
Rabbit monoclonal anti- α-tubulin	Caballero-Camino FJ *et al*. Hepatology 2020.	Abcam	ab52866	EP1332Y
Rabbit monoclonal IgG	Liu S *et al*. Tumour Biol 2014	Abcam	ab172730	EPR25A
Anti-mouse IgG, HRP-linked Antibody	Uribarri AD *et al*. Gut 2015.	Cell Signaling	#7076	X
Anti-rabbit IgG, HRP-linked Antibody	Yang, D *et al*. Blood 2003.	Cell Signaling	#7074	X

1.2 Cell lines

Name	Citation	Supplier	Cat no.	Passage no.	Authentication test method
Normal Human Cholangiocytes (NHC)	Uribarri AD *et al*. Gut 2015. Banales JM *et al*. Hepatology 2012.	X	X	P4-P10	Biliary markers expression (CK-7, CK-19, AQP, AE2, CFTR)
GANAB mutated human cholangiocytes (ADPKD)	Uribarri AD *et al*. Gut 2015. Banales JM *et al*. Hepatology 2012.	X	X	P4-P10	Biliary markers expression (CK-7, CK-19, AQP, AE2, CFTR)

Normal rat cholangiocytes (NRC)	Vroman B *et al.* Laboratory Investigation 1996.	X	X	P19-P25	Biliary markers expression (CK-7, CK-19, AQP, AE2, CFTR)
Pkhd1 mutated rat cholangiocytes (PCK)	Vroman B *et al.* Laboratory Investigation 1996.	X	X	P19-P25	Biliary markers expression (CK-7, CK-19, AQP, AE2, CFTR)

1.3 Organisms

Name	Citation	Supplier	Strain	Sex	Age	Overall n number
Sprague Dawley (SD)	Mason SB *et al*. The Anatomical Record 2011 Masyuk TV *et al*. The American Journal of pathology 2004.	Charles River Laboratories Inc.	Crl:CD(SD)	Male	8 weeks	4
Sprague Dawley (SD)	Mason SB *et al*. The Anatomical Record 2011 Masyuk TV *et al*. The American Journal of pathology 2004.	Charles River Laboratories Inc.	Crl:CD(SD)	Female	8 weeks	4
PCK	Mason SB *et al*. The Anatomical Record 2011 Masyuk TV *et al*. The American Journal of pathology 2004.	Charles River Laboratories Inc.	PCK/CrljCrl-*Pkhd1*^{pck}/Crl	Male	8 weeks	13
PCK	Mason SB *et al*. The Anatomical Record 2011 Masyuk TV *et al*. The American Journal of pathology 2004.	Charles River Laboratories Inc.	PCK/CrljCrl-*Pkhd1*^{pck}/Crl	Female	8 weeks	13

1.4 Sequence based reagents

Name	Sequence	Supplier
ATF6	Forward: 5'-GCTGGATGAAGTTGTGTCAGAG-3'<br>Reverse: 5'-TGTTCCAACATGCTCATAGGTC-3'	Sigma-Aldrich
ATG5	Forward: 5'-CGTCCTGTGGCTGCAGATG-3'<br>Reverse: 5'-AAGGACAAACTTCTTTGAGGAGA-3'	Sigma-Aldrich
BAX	Forward: 5'-CCCGAGAGGTCTTTTTCCGA-3'<br>Reverse: 5'-CCAGCCCATGATGGTTCTGAT-3'	Sigma-Aldrich
BECN1	Forward: 5'-GATGGAAGGGTCTAAGACGTCCAA-3'<br>Reverse: 5'-TTTCGCCTGGGCTGTGGTAAG-3'	Sigma-Aldrich
BIM	Forward: 5'-GCCCCTACCTCCCTACAGAC-3'	Sigma-Aldrich

	Reverse: 5'-CCTTATGGAAGCCATTGCAC-3'	
CHOP	Forward: 5'-TCTTCATACATCACCACACC-3'<br>Reverse: 5'-CTTGTGACCTCTGCTGGTTC-3'	Sigma-Aldrich
DR5	Forward: 5'-ACAGTTGCAGCCGTAGTCTTG-3'<br>Reverse: 5'-CCAGGTCGTTGTGAGCTTCT-3'	Sigma-Aldrich
GAPDH	Forward: 5'-CCAAGGTCATCCATGACAAC-3'<br>Reverse: 5'-TGTCATACCAGGAAATGAGC-3'	Sigma-Aldrich
GRP78	Forward: 5'-GAGCTGTGCAGAAACTCCGGCG-3'<br>Reverse: 5'-ACCACCTGCTGAATCTTTGGAATTCGAGT- 3'	Sigma-Aldrich
IRE1α	Forward: 5'-AGGGACAGGAGGGAATCGTA-3'<br>Reverse: 5'-CAGTCCCTAATGCCACACCT-3'	Sigma-Aldrich
LC3B	Forward: 5'-GGTGAGAAGCAGCTTCCTGT-3'<br>Reverse: 5'-TCTCCTGGGAGGCATAGACC-3'	Sigma-Aldrich
MAT1A	Forward: 5'-GTTCACATCGGAGTCTGTGG-3'<br>Reverse: 5'-GATGTGCTTGATGGTGTCC-3'	Sigma-Aldrich
MAT2A	Forward: 5'-GCTACGAGTAGAACGCTGTC-3'<br>Reverse: 5'-GCATCAGGATCCTGCTGAAG-3'	Sigma-Aldrich
MAT2B	Forward: 5'-CAGATGCTGCCTCTCAAC-3'<br>Reverse: 5'-GAACCTCTGCTGCCAGTG-3'	Sigma-Aldrich
SQSTM1/P62	Forward: 5'-TACGACTTGTGTAGCGTCTG-3'<br>Reverse: 5'-CGTGTTTCACCTTCCGGAG-3'	Sigma-Aldrich
PERK	Forward: 5'-CAGGCAAAGGAAGGAGTCTG-3'<br>Reverse: 5'-AACAACTCCAAAGCCACCAC-3'	Sigma-Aldrich
SAE1	Forward: 5'-CTTGCTGCTCCAGGGATGTC-3'<br>Reverse: 5'-GACCATCGTTGTCTCAGAAG-3'	Sigma-Aldrich
SOX9	Forward: 5'-GTACCCGCACTTGCACAAC-3'<br>Reverse: 5'-TCGCTCTCGTTCAGAAGTCTC-3'	Sigma-Aldrich
SOX17	Forward: 5'-GTGGACCGCACGGAATTTG-3'<br>Reverse: 5'-GGAGATTCACACCGGAGTCA-3'	Sigma-Aldrich
SUMO1	Forward: 5'-CCGTCATCATGTCTGACC-3'<br>Reverse: 5'-GGAACACCCTGTCTTTGAC-3'	Sigma-Aldrich
UBA2	Forward: 5'-CGTTGCCTACCATGACAGC-3'<br>Reverse: 5'-GAGGAACATCAGCTGCCAG-3'	Sigma-Aldrich
Ubiquitin	Forward: 5'-CCTGAGGGGTGGCTGTTA-3'<br>Reverse: 5'-GCTACCATGCAACGAAACC-3'	Sigma-Aldrich
UBE2I	Forward: 5'-GGACTTTGAACATGTCGG-3'<br>Reverse: 5'-CCGAAGGGTACACATTCGG-3'	Sigma-Aldrich
s-XBP1	Forward: 5'-GCTGAGTCCGCAGCAGGT-3'<br>Reverse: 5'-CTGGGTCCAAGTTGTCCAGAAT-3'	Sigma-Aldrich
ZO-1	Forward: 5'-CGGTCCTCTGAGCCTGTAAG-3'<br>Reverse: 5'-GGATCTACATGCGACGACAA-3'	Sigma-Aldrich
α-Sma	Forward: 5'-CGCCATCAGGAACCTCGAGAAG-3'<br>Reverse: 5'-ATCATCACCAGCAAAGCCCG-3'	Sigma-Aldrich
Col1a1	Forward: 5'-GACTGTCCCAACCCCCAAA-3’<br>Reverse: 5'-CTTGGGTCCCTCGACTCCTA-3’	Sigma-Aldrich
Ctgf	Forward: 5'-CTAGCTGCCTACCGACTGGA-3'<br>Reverse: 5'-GCCCATCCCACAGGTCTTAG-3'	Sigma-Aldrich
Cxcl1 (IL8 homolog)	Forward: 5'-ACTCAAGAATGGTCGCGAGG-3'<br>Reverse: 5'-ACGCCATCGGTGCAATCTAT-3'	Sigma-Aldrich
Gapdh	Forward: 5'-TGTGAACGGATTTGGCCGTA-3'<br>Reverse: 5'-ATGAAGGGGTCGTTGATGGC-3'	Sigma-Aldrich
Il6	Forward: 5'-CATTCTGTCTCGAGCCCACC-3'<br>Reverse: 5'- GTCCCAAGAAGGCAACTGG-3'	Sigma-Aldrich
Mat1a	Forward: 5'-CGAGAAGTGTGACACCATG-3'<br>Reverse: 5'-CATCCAGCACTGCATCAC-3'	Sigma-Aldrich
Mat2a	Forward: 5'-CACTTCAGAGTCTGTAGG-3'<br>Reverse: 5'-CCTGCACCAATGTCTTCCTC-3'	Sigma-Aldrich
Mat2b	Forward: 5'-GAGAAGGAGCTCTCCATCC-3'<br>Reverse: 5'-CAGCAGGTTCACCTGTTCG-3'	Sigma-Aldrich
Sae1	Forward: 5'-GTCGACCAGATCTGTCACAG-3'<br>Reverse: 5'-CACTCCAGTCCACTGCTAG-3'	Sigma-Aldrich
Sumo1	Forward: 5'-GACAGCAGTGAGATCCAT-3'<br>Reverse: 5'-CTAAGAGATGGAGTGCCAG-3'	Sigma-Aldrich
Tgfβ1	Forward: 5'-CTGCTGACCCCCACTGATAC-3'<br>Reverse: 5'-GGAGATTCACACCGGAGTCA-3'	Sigma-Aldrich

Uba2	Forward: 5'-CCATGACAGCATCATGAACC-3'<br>Reverse: 5'-CTCTGTCAGGAGACACTTC-3'	Sigma-Aldrich
Ube2i	Forward: 5'- CGTGTATCCTTCTGGCAC-3'<br>Reverse: 5'-CTTCGCTTGTGCTCGAAC-3'	Sigma-Aldrich

1.5 Biological samples

Description	Source	Identifier
Cystic wall biopsies from patients with ADPLD (*PRKCSH* and *SEC63* mutated)	Radboud University Medical Center (Nijmegen, The Netherlands)	2012/317
Healthy human gallbladder biopsies obtained from metastatic colon cancer patients	Donostia University Hospital (San Sebastian, Spain)	MSA-MMR-2017-01
Healthy human liver biopsies obtained from metastatic colon cancer patients	Donostia University Hospital (San Sebastian, Spain)	MSA-MMR-2017-01

1.6 Deposited data

Name of repository	Identifier	Link
DAVID	DAVID 6.8	https://david.ncifcrf.gov/summary.jsp
Heatmapper	Heatmapper	http://heatmapper.ca/
STRING	STRING 11.0	https://string-db.org/

1.7 Software

Software name	Manufacturer	Version
Bio-Rad CFX Maestro™ 1.0	Bio-Rad	4.0.2325.0418
Microsoft Excel for Mac	Microsoft Corporation	16.32
Guava ® InCyte™	Merck Millipore	3.1.1
i-control™	Tecan	1.8
NIS-Element AR	Nikon	3.2
Image J	National Institutes of Health-Bethesda	1.50
GraphPad Prism	GraphPad Software, Inc.	8.3.0
PEAKS Studio	Bioinformatics Solutions Inc	8.5
Proteome Discoverer	Thermo Fisher	1.4
DataAnalysis	Bruker	
ZEN	Zeiss	3.2 (blue edition)

1.8 Other (e.g. drugs, proteins, vectors etc.)

S-adenosyl-l-methionine disulfate p-toluenesulfonate (SAMe)	Gnosis S.p.A.	
MG132	Sigma Aldrich	
UBE2I shRNA	Sigma Aldrich	TRCN0000329448
Scramble shRNA	Sigma Aldrich	

1.9 Please provide the details of the corresponding methods author for the manuscript:

Jesus M. Banales, PhD, Department of Liver and Gastrointestinal Diseases, Biodonostia Health Research Institute – Donostia University Hospital, Paseo del Dr. Begiristain s/n, E-20014, San Sebastian, Spain. e-mail: jesus.banales@biodonostia.org / Phone: +34 943006067; Fax: +34 943006250
or
Joost P.H. Drenth, M.D., Ph.D., Department of Gastroenterology and Hepatology, Radboud University Medical Center, PO Box 9101, 6500 HB Nijmegen, The Netherlands. E-mail: joost.drenth@radboudumc.nl / Phone: +31 243616999; Fax: +31 24 363 5129

2.0 Please confirm for randomised controlled trials all versions of the clinical protocol are included in the submission. These will be published online as supplementary information.

