# Supplementary material for: Targeting UBC9-mediated protein hyper-SUMOylation in cystic cholangiocytes halts polycystic liver disease in experimental models
Source: J Hepatol. Author manuscript; Available in PMC 2021 May 27. (PMC8157180; doi:10.1016/j.jhep.2020.09.010)
Supplement: Suppl Material 1 [file NIHMS1666894-supplement-Suppl_Material_1.pdf]

Targeting UBC9-mediated Protein Hyper-SUMOylation in Cystic Cholangiocytes Halts Polycystic Liver Disease in Experimental Models

Pui Y. Lee-Law,[1,2*] Paula Olaizola,[1*] Francisco J. Caballero-Camino,[1] Laura Izquierdo-Sánchez,[1,3] Pedro M. Rodrigues,[1] Alvaro Santos-Laso,[1] Mikel Azkargorta,[3,4] Felix Elortza,[3,4] Maria L. Martinez-Chantar,[3,5] Maria J. Perugorria,[1,3] Patricia Aspichueta,[6,7] Marco Marzioni,[8] Nicholas F. LaRusso,[9] Luis Bujanda,[1,3] Joost P.H. Drenth,[2,$] and Jesus M. Banales.[1,3,10,$]

Table of contents

Materials and Methods 1

Supplementary Figures 9

Supplementary Tables 20

References 32

Materials and Methods

RNA isolation and gene expression

RNA isolation was performed in human and rat liver samples and cell culture using TRI Reagent (Sigma). Subsequently, 1 µg of RNA isolated from human liver samples were reverse transcribed using the SuperScript VILO cDNA Synthesis Kit (Invitrogen) in a Veriti Thermal Cycler (Applied Biosystems). On the other hand, cells and rat liver samples were reverse transcribed using a mixture of DNAse I, Amplification Grade (Invitrogen), M-MLV Reverse Transcriptase (Invitrogen), RNaseOUT Recombinant Ribonuclease Inhibitor (Invitrogen), Random Primers (Invitrogen) and illustra Solution dNTPs (GE Life Sciences). The gene expression (mRNA) of specific primers sequences (Supplementary CTAT Table and Supplementary Table 2) was determined by real-time quantitative polymerase chain reaction (qPCR) using iQ SYBR Green Supermix (Bio-Rad) in a CFX96 Touch Real-Time PCR Detection System as previously described.[1]

Animals and treatment

The well-defined PCK rat (commercially available by Charles River Laboratory) carries a spontaneous splicing mutation (IVS35-2A→T) in the *polycystic kidney and hepatic disease 1* (*PKHD1*) orthologous gene, responsible for an ARPKD/congenital hepatic fibrosis phenotype.[2] The PCK rat represents an accurate and useful animal model of slowly progressive PLD phenotype.[3] The liver phenotype in PCK rats is characterized by extensive cyst formation, as the majority disconnect from the bile ducts throughout time, and hepatomegaly with unaltered liver parenchyma.[4] Hepato-renal cystogenesis and fibrosis gradually develop after 8 weeks of age in both male and female PCK rats, and concurrently elevated serum levels of blood urea nitrogen, creatinine, bilirubin, cholesterol,

triglycerides and alkaline phosphatase are observed.[3] Although the pathologies become evident after 25 weeks of age, there is considerable variability.

In this study, PCK rats (8 weeks of age) were grouped in: 1) non-treated (n=14) and 2) treated (n=12) with S-adenosyl-l-methionine disulfate p-toluenesulfonate (SAMe, kindly provided by Gnosis S.p.A., Derio, Italy). Every group had 50% male and 50% female PCK rats, as published studies demonstrated no significant differences between both sexes (Supplementary Figure 1).[5–8] SAMe dosage was set at 20 mg/kg/day, as published clinical trials in liver diseases showed no severe adverse events.[9,10] SAMe dissolved easily in drinking water of the animals, thus based on daily weight monitoring, SAMe was administered by oral gavage with an orogastric canula for 5 months (on a 5-day-on/2-day-off schedule). As negative control of the disease, eight-weeks old wild-type (Sprague-Dawley, Charles River Laboratory) rats (n=8) were maintained in parallel. During the entire study, all animals were weighed weekly, and had *ad libitum* access to food (standard diet) and water.

At the beginning, and after 6 weeks, 12 weeks and at the sacrifice, blood samples were collected from every animal. Levels of serum biochemical markers such as alkaline phosphatase (ALP), aspartate aminotransferase (AST), alanine aminotransferase (ALT), albumin, blood urea and total protein were measured. For every blood collection, the animals were anesthetized with isoflurane (2.5% in inhaling oxygen, at a flow of 0.3 L/min). Non-treated and treated rats were sacrificed after 5 months, and blood, bile, liver, kidneys and spleen were collected. The harvested tissues were stained with hematoxylin/eosin or picrosirius red, for measuring hepatorenal cysts and liver fibrosis, respectively.

Hepatorenal cystogenesis and liver fibrosis were quantitated using ImageJ software (National Institutes of Health, USA).[11]

All animal experimental procedures were approved by the *Animal Experimentation Ethics Committee* of Biodonostia Health Research Institute (CEEA17/007).

Immunoblotting

Whole cells lysates of cultured human cholangiocytes were extracted with radio-immunoprecipitation (RIPA) lysis buffer and freshly prepared 20 mM N-ethylmaleimide (NEM, isopeptidase inhibitor preventing protein deSUMOylation).[12] Changes in small ubiquitin like modifier 1 (SUMO1) and SUMO1-conjugated proteins, UBC9, acetylated α-tubulin, p62 and ubiquitin-conjugated proteins were analyzed by western blotting (WB) using protein from cell extracts, which were separated in a 12.5% sodium dodecyl sulfate polyacrylamide gel electrophoresis (SDS-PAGE), and electro-transferred to a nitrocellulose membrane (Bio-Rad). After blocking with 5% skim milk powder/TBS-0.1%Tween, membranes were incubated with the relevant primary antibody at 1:1000 dilution (Supplementary CTAT table and Supplementary Table 3) overnight at 4ºC. Then, membranes were probed with an appropriate horseradish peroxidase-conjugated secondary antibody at 1:5000 dilution for 1 hour at room temperature. Using Novex ECL Chemiluminescent Substrate Reagent Kit (Invitrogen) and iBright CL1500 Imaging System (Invitrogen), bands were visualized and quantification was carried out with ImageJ software (National Institutes of Health, USA).[11] β-actin or α-tubulin protein levels were used to normalize protein loading and expression.

Cell viability, proliferation and apoptosis

Cell viability was assessed by using the colorimetric WST-1 (Roche) assay, as per manufacturer's instructions. Briefly, $3x10^3$ cells were seeded in each well of a collagen-coated 96-well plate in fully-supplemented Dulbecco's modified Eagle's medium (DMEM)/F-12 medium.[13] The day after, cells were treated with different dosages of SAMe for 48 hours.

Cell proliferation was measured by flow-cytometry using CellTrace CFSE Cell Proliferation Kit (Invitrogen), following manufacturer's instructions. In short, cells were harvested, resuspended with PBS 0.1% BSA and labeled with 10 μM CFSE. Then, cold fully-supplemented DMEM/F-12 medium was added and the cells were placed on ice for 5 min. After three washes with fully-supplemented DMEM/F-12 medium, $3x10^4$ cells were seeded per well in a 12-well plate. The next day, cells received different dosages of SAMe for 48 hours. Fluorescence of cells was measured by flow-cytometry in Guava easyCyte 8HT Benchtop Flow Cytometer (Merck Millipore) after harvesting, centrifuging and resuspending the cells in PBS.

Apoptotic rate of cells was evaluated by flow-cytometry Shortly, $2.5x10^4$ cells per well were seeded in a 24-well plate in fully-supplemented DMEM/F-12 medium. Twenty-four hours later, cells were treated with different dosages of SAMe and/or MG132 for 48 hours. Next, cells were harvested and stained with FITC Annexin V (BioLegend) and TO-PRO-3 Iodide (Invitrogen). Fluorescence was measured by flow-cytometry in Guava easyCyte 8HT Benchtop Flow Cytometer (Merck Millipore).

Primary cilia

Polycystic human cholangiocytes (PHC) were seeded at a density of $5x10^4$ and cultured on collagen-coated glass coverslips in a 24-well plate until they reached confluence. Afterwards, SAMe (1.0 mM) was administered and replaced every 48 hours for a total period of 7 days. For the analysis of the number of ciliated cholangiocytes, cells were washed with PBS 1X and fixed with ice-cold methanol for 10 min at -20°. Samples were washed 3 times with PBS 1X and incubated with 0.5% Triton-100X/PBS 1X for 20 min at room temperature for cell membrane permeabilization. Next, cells were blocked with 1%BSA/5%FBS/0.5% Triton-100X/PBS 1X for 30 min at room temperature and incubated overnight at 4°C with acetylated α-tubulin antibody (1:1000; Sigma). Afterwards, cells were incubated for 2 hours at room temperature with Alexa Fluor 488 secondary antibody (Life Technologies). Coverslips were mounted on slides using VECTASHIELD® mounting medium with DAPI (Vector laboratories). Images were obtained at 40X with a confocal laser scanning microscope (LSM 900, ZEISS). Finally, images were analyzed and the number of ciliated cells was calculated.

Cell transfection with short hairpin RNA

PHC were seeded in a 6-well plate at a density of $2x10^5$ and left for overnight attachment before transfection. Then, DNA plasmids of control short hairpin RNAs (shRNA, Sigma) or shRNA against *ubiquitin conjugating enzyme E2 I (UBE2I)* (TRCN0000329448, Sigma) in Opti-MEM (Gibco) were combined with FuGENE HD (Promega) at a ratio of 1:4 in order to form complexes. After 15 min of incubation, the mixture was added dropwise to the wells. Medium was changed after 24 hours to fully-supplemented DMEM/F-12 medium. In order to obtain stable transfected cell lines, 1.5 μg/ml puromycin (Sigma) was added to the cells

for 7 days. *UBE2I* knockdown was verified by protein expression using immunoblotting.

Immunoprecipitation

First, 1 mg of whole cell lysate from normal human cholangiocytes (NHC) and PHC was precleared by incubating the samples with Dynabeads Protein G (Invitrogen), concentration according to manufacturer's instructions. In parallel, another amount of Dynabeads Protein G was incubated with SUMO1 antibody (Abcam) or, as negative control, with immunoglobulin G (IgG) antibody (Abcam) and crosslinked with freshly prepared bis(sulfosuccinimidyl)suberate (BS^3, Thermo Scientific). Then, the precleared cell lysates were incubated with the SUMO1- or IgG- crosslinked Dynabeads. Finally, the proteins were eluted from the Dynabeads with 2% SDS.

Mass spectrometry and proteomic analysis

Samples of control shRNA and shRNA against *UBE2I*, as well as SUMO1-immunoprecipitation (IP) lysates, were extracted or eluted using 7 M urea, 2 M thiourea, 4% CHAPS. Samples were incubated for 30 min at room temperature under agitation and digested following the Filter-Aided Sample Preparation protocol [14] with minor modifications. Trypsin was added to a trypsin:protein ratio of 1:10, and the mixture was incubated overnight at 37°C, dried out in a RVC2 25 speedvac concentrator (Christ), and resuspended in 0.1% formic acid (FA).

Samples were analyzed in a novel hybrid trapped ion mobility spectrometry – quadrupole time of flight mass spectrometer parallel accumulation serial fragmentation (tims TOF Pro with PASEF, Bruker Daltonics) coupled online to a nanoElute liquid chromatograph (Bruker). This mass spectrometer takes advantage of a novel scan mode termed parallel accumulation – serial

fragmentation (PASEF), which multiplies the sequencing speed without any loss in sensitivity [15] and has been proven to provide outstanding analytical speed and sensibility for proteomics analyses.[16] Sample (200 ng) was directly loaded in a 15 cm nanoElute FIFTEEN C18 analytical column (Bruker) and resolved at 400 nl/min with a 30 min gradient. Column was heated to 50ºC using an oven.

Protein identification and quantification for control shRNA and shRNA against *UBE2I* samples were carried out using PEAKS software (Bioinformatics Solutions Inc). Searches were carried out against a database consisting of human entries (Uniprot/Swissprot), with precursor and fragment tolerances of 20 ppm and 0.05 Da. Only proteins identified with at least two peptides at false discovery rate (FDR) <1% were considered for further analysis. Data was loaded onto Perseus platform [17] and further processed (log2 transformation, imputation).

Protein identification and quantification for SUMO-1 IP lysates were carried out using Mascot search engine (Matrix Science Ltd.) through Proteome Discoverer software 1.4 (Thermo). 50ppm and 0.05Da were used for precursor and fragment searches, and a database consisting of human entries (Uniprot/Swissprot) was used for the searches. Only proteins identified with at least two peptides at FDR<1% in at least two sample replicas and not present in the negative control were considered for further analysis.

Spectral counts for each protein (the number of identified spectra matching to peptides from that protein, also named SpC or PSMs) were used for the differential analysis. Data was loaded onto Perseus platform and further processed (log2 transformation, imputation). A *t*-test was applied in order to determine the statistical significance of the differences detected. Functional

analyses of proteins were performed in the STRING database,[18] and by gene ontology (GO) enrichment using DAVID Bioinformatics Resources. Details are outlined in the Supplementary data.[19] In addition, heatmaps were generated using Heatmapper for data visualization.[20] The mass spectrometry proteomics data have been deposited to the ProteomeXchange Consortium via the PRIDE [21] partner repository with the dataset identifiers PXD021139 and PXD021140.

Supplementary Figures

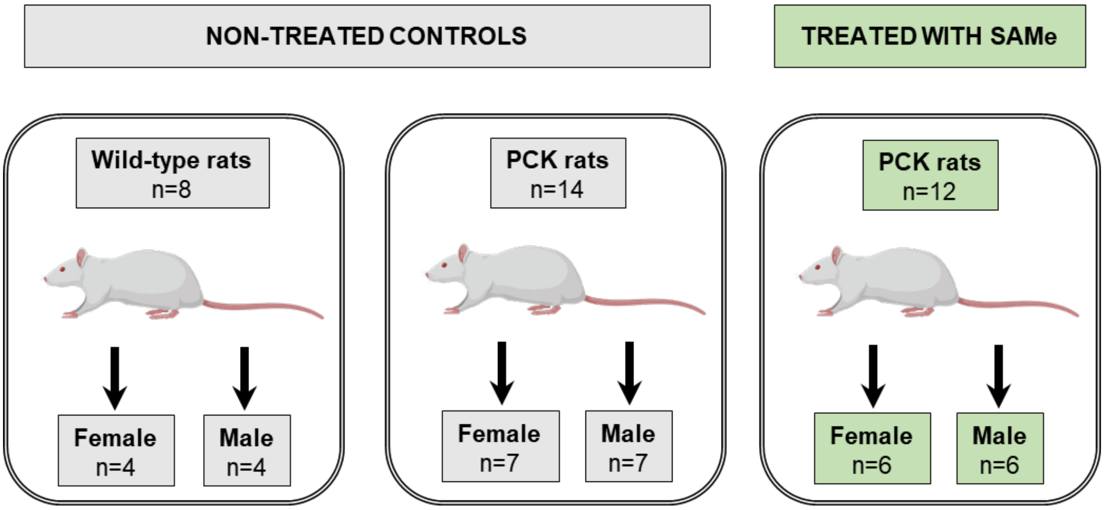

Supplementary Fig. 1. Each group of this study consisted 50% female and 50% male rats.

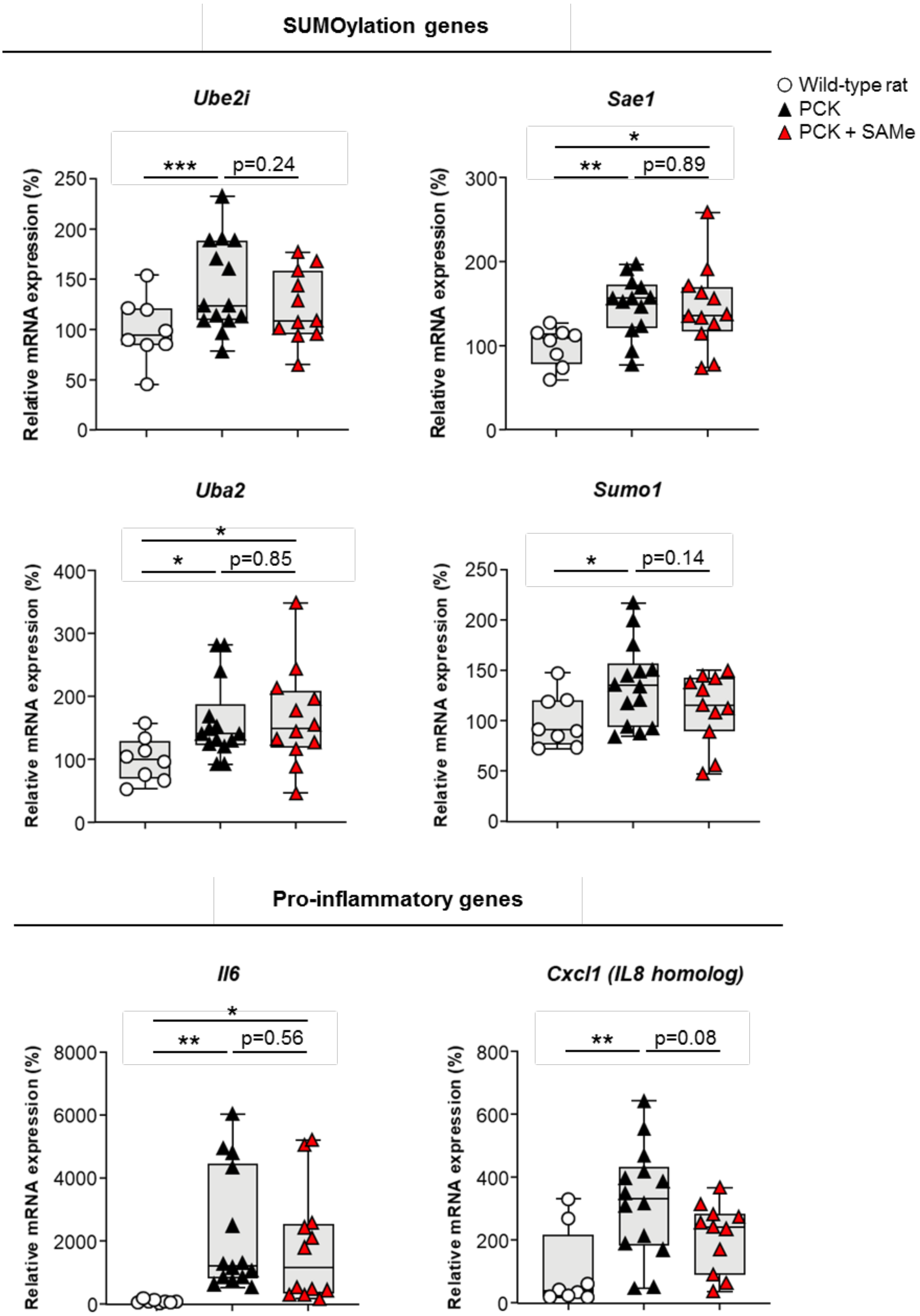
SUMOylation genes
Ube2i
Sae1
Uba2
Sumo1
Pro-inflammatory genes
Il6
Cxcl1 (IL8 homolog)
Wild-type rat
PCK
PCK + SAMe
Relative mRNA expression (%)
***
p=0.24
*
**
p=0.89
p=0.85
p=0.14
p=0.56
p=0.08

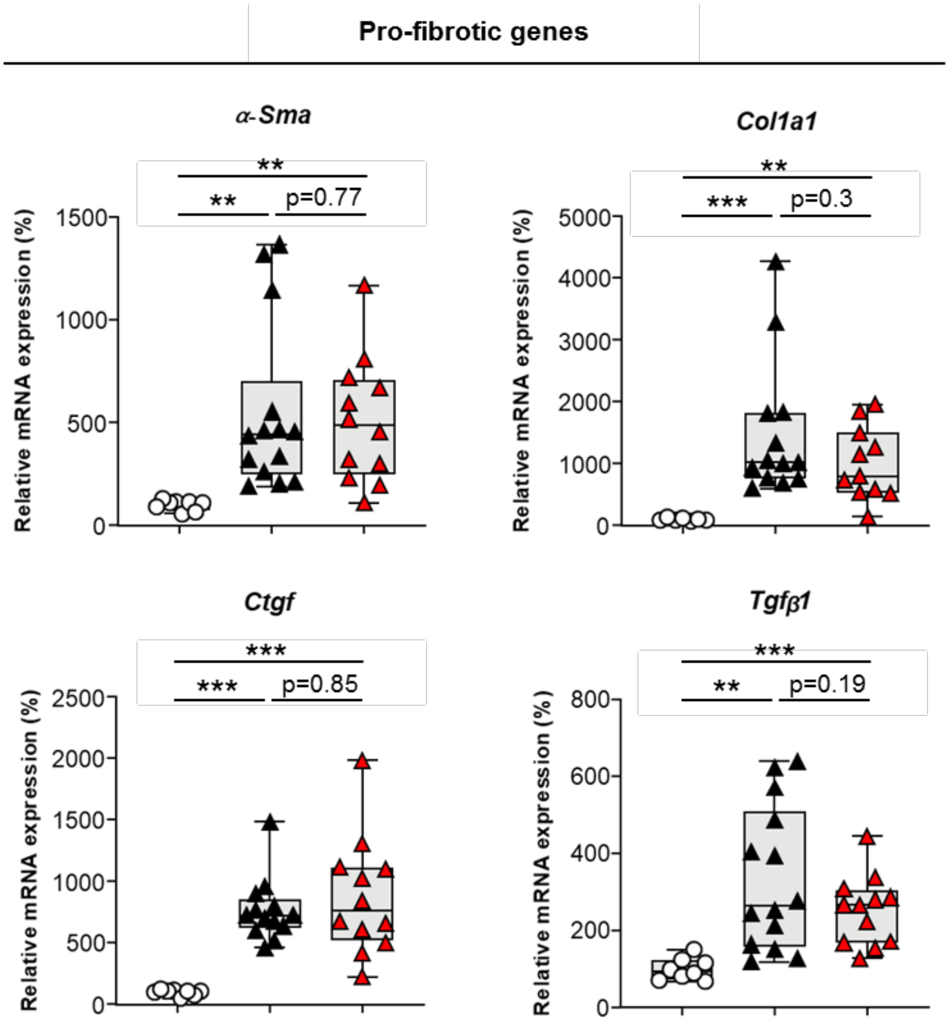

Supplementary Fig. 2. mRNA levels of SUMOylation, pro-inflammatory and profibrotic genes in whole liver tissue samples from wild-type, non-treated PCK and SAMe-treated PCK rats. Five months of SAMe treatment (20 mg/kg/day). Data shown as mean ± SEM. *p<0.05; **p<0.01; ***p<0.001 (one-way ANOVA, Kruskal-Wallis tests)

Abbreviations: *α-Sma*, alpha smooth muscle actin; *Col1a1*, collagen type 1 alpha 1 chain; *Ctgf*, connective tissue growth factor; *Cxcl1*, chemokine (C-X-C motif) ligand 1; *Il6*, interleukin 6; *Sae1*, SUMO1 activating enzyme subunit 1; *Sumo1*, small ubiquitin like modifier 1; *Tgfβ1*, transforming growth factor beta 1; *Uba2*, SUMO1 activating enzyme subunit 2; *Ube2i*, ubiquitin conjugating enzyme E2 I.

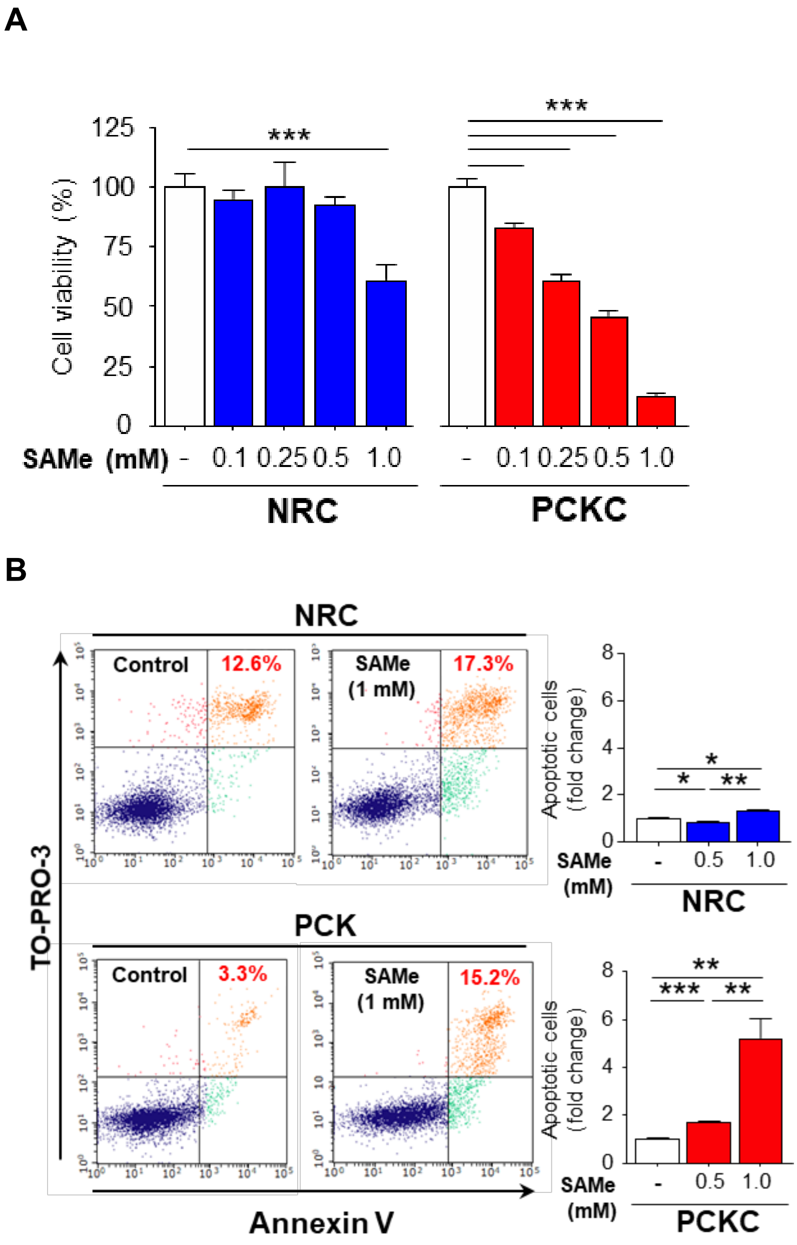
A
125
100
75
50
25
0
Cell viability (%)
***
***
SAMe (mM)
-
0.1
0.25
0.5
1.0
NRC
PCKC
B
NRC
Control
12.6%
SAMe
(1 mM)
17.3%
PCK
Control
3.3%
15.2%
TO-PRO-3
Annexin V
Apoptotic cells
(fold change)
*
**
***
PCKC

C

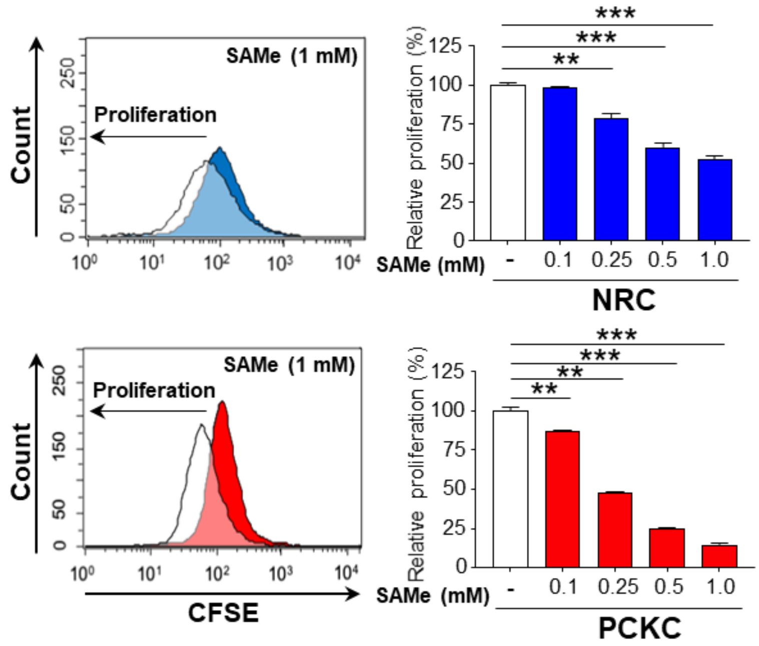

Supplementary Figure 3. Cell (A) viability (n=6), (B) apoptosis (n=4), and (C) proliferation (n=3) in untreated or SAMe-treated NRC and PCKC. Data shown as mean + SEM. *$p<0.05$; **$p<0.01$; ***$p<0.001$ (one-way ANOVA, Kruskal-Wallis, Mann-Whitney or 2-tailed t-tests).

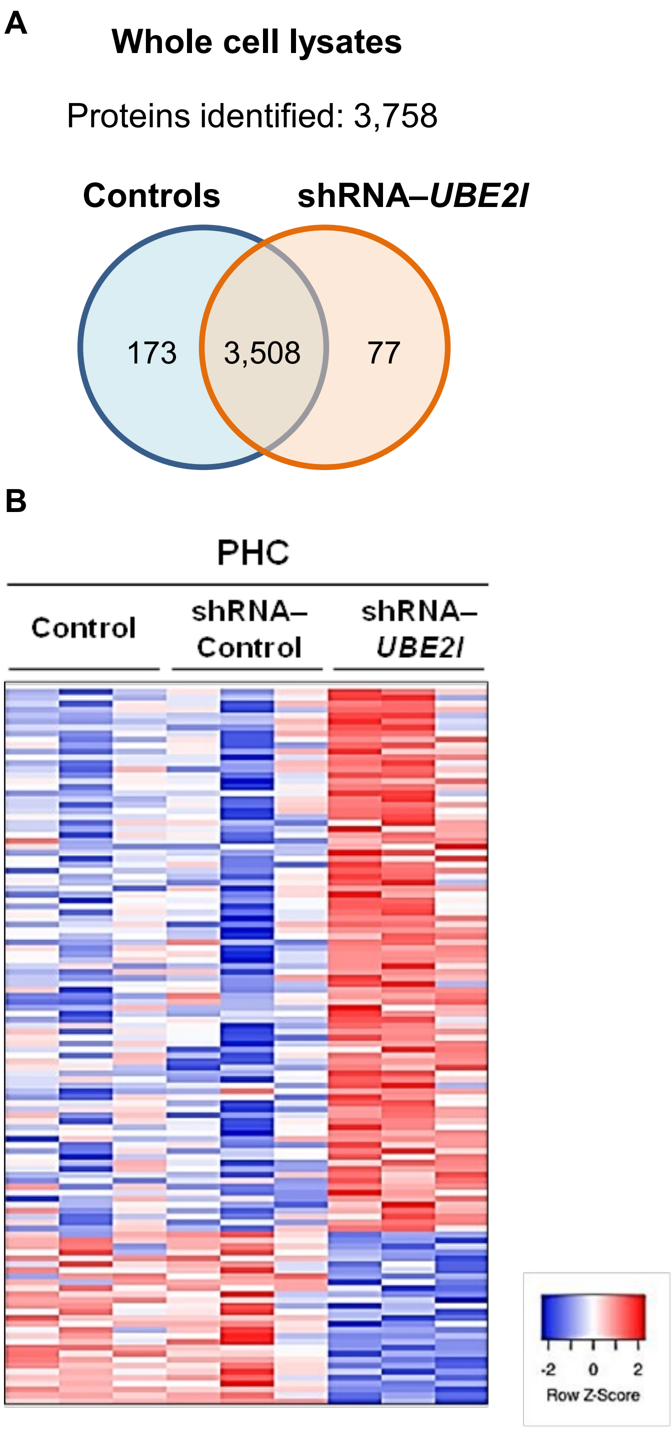
A
Whole cell lysates
Proteins identified: 3,758
Controls
shRNA–UBE2I
173
3,508
77
B
PHC
Control
shRNA–
Control
shRNA–
UBE2I
-2
0
2
Row Z-Score

C

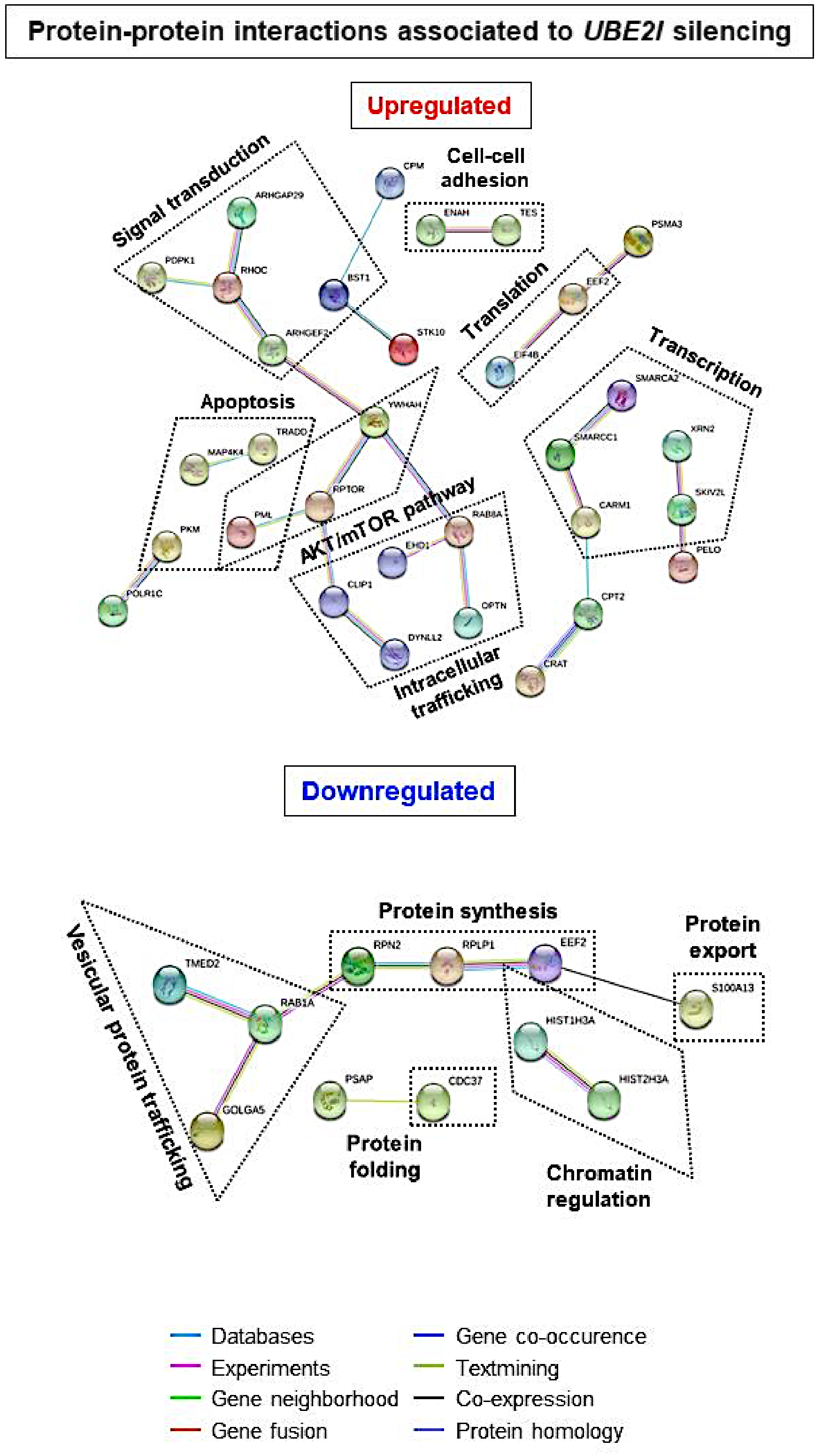

Supplementary Figure 4. (A) Venn diagram of all identified proteins (3,758). (B) Proteomic analyses of the significant dysregulated proteins (n=120) by heatmap representation, and (C) PPI network associated to *UBE2I* silencing.

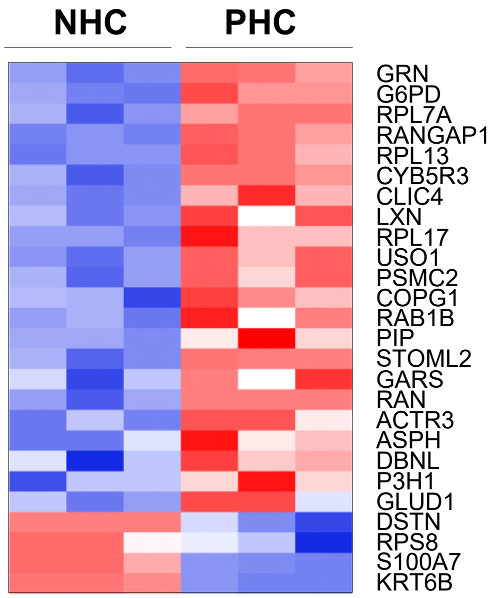

Supplementary Fig. 5. Heatmap including the significantly enriched SUMO1-IP proteins in PHC compared to NHC.

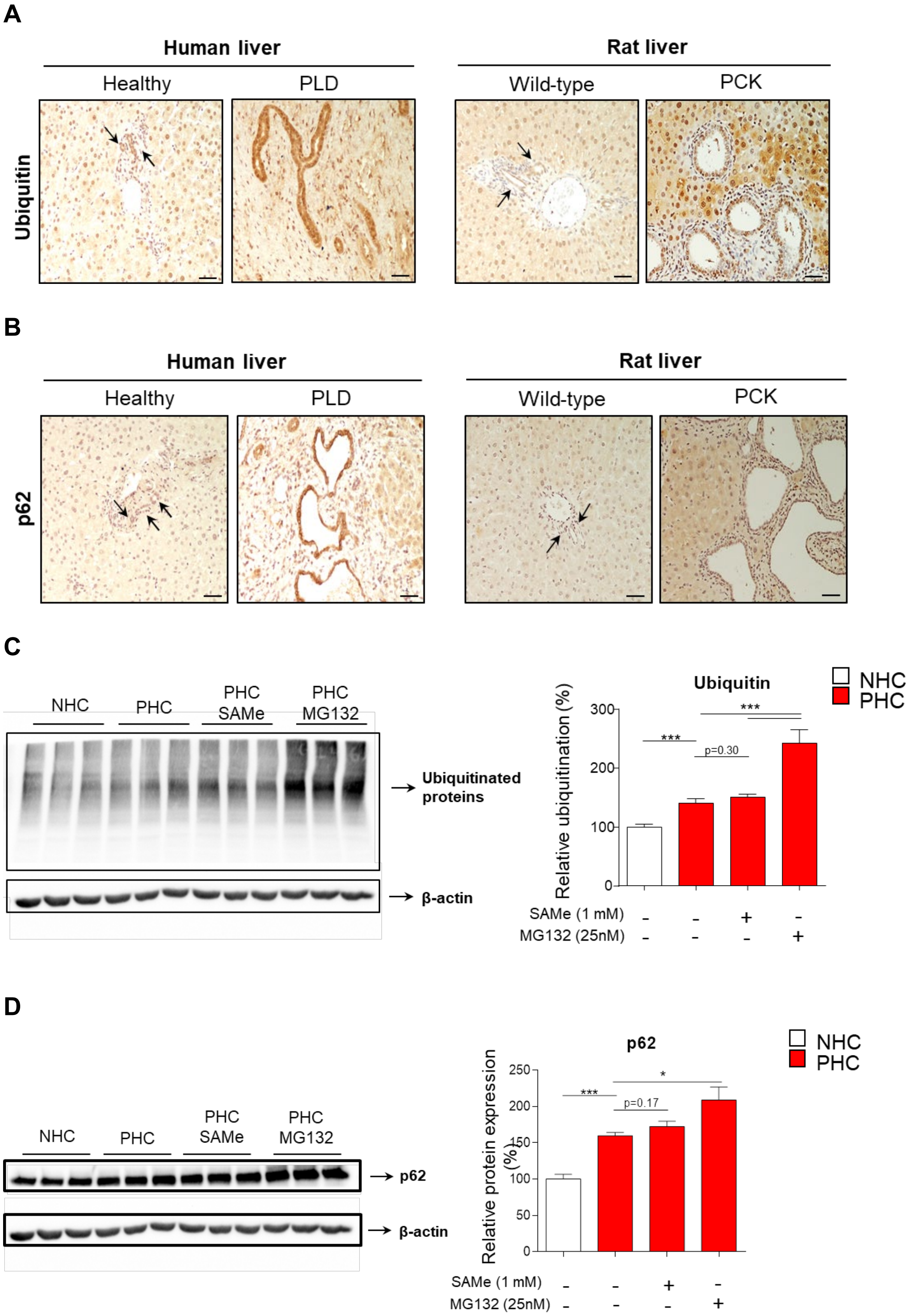
A
Human liver
Rat liver
Healthy
PLD
Wild-type
PCK
Ubiquitin
B
Human liver
Rat liver
Healthy
PLD
Wild-type
PCK
p62
C
NHC
PHC
PHC SAMe
PHC MG132
Ubiquitinated proteins
β-actin
Ubiquitin
NHC
PHC
Relative ubiquitination (%)
***
***
p=0.30
SAMe (1 mM)
MG132 (25nM)
D
NHC
PHC
PHC SAMe
PHC MG132
p62
β-actin
p62
NHC
PHC
Relative protein expression (%)
***
*
p=0.17
SAMe (1 mM)
MG132 (25nM)

E

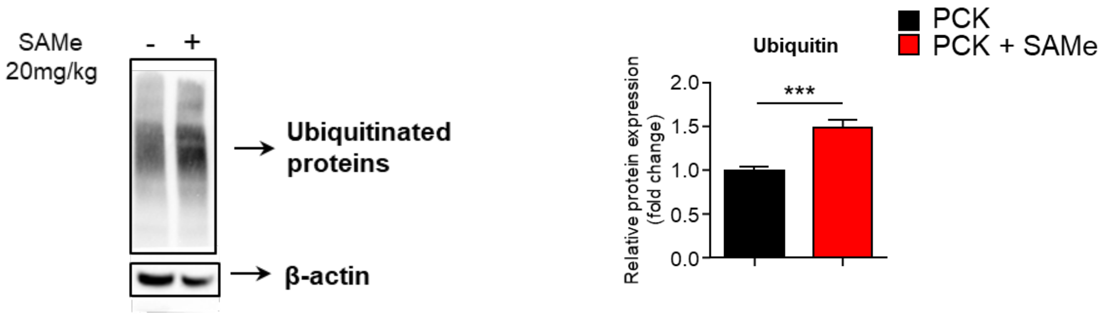

F

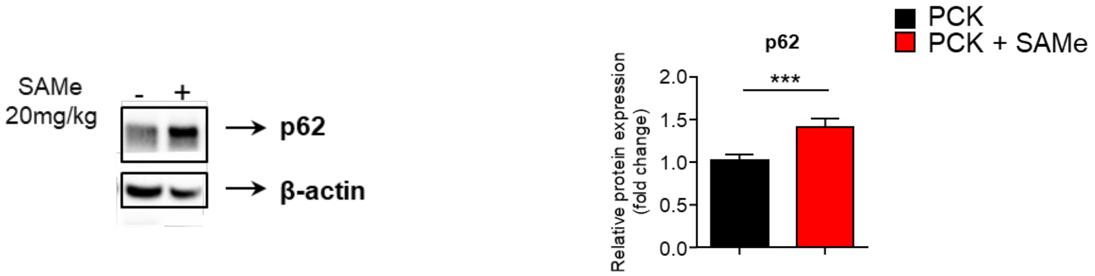

Supplementary Fig. 6. A) Representative IHC images of ubiquitin and B) p62 in human and rat liver tissue. C) Representative immunoblot and quantification of ubiquitin-conjugated proteins and D) p62 expression in NHC and PHC under baseline conditions, as well as in PHC under SAMe or MG132 incubation (n=3). E) Representative immunoblot and quantification of ubiquitin-conjugated proteins and F) p62 expression in non-treated (n=14) or SAMe-treated (n=12) PCK livers. Data shown as mean + SEM. *p<0.05; ***p<0.001 (one-way ANOVA, Kruskal-Wallis, 2-tailed t-tests).

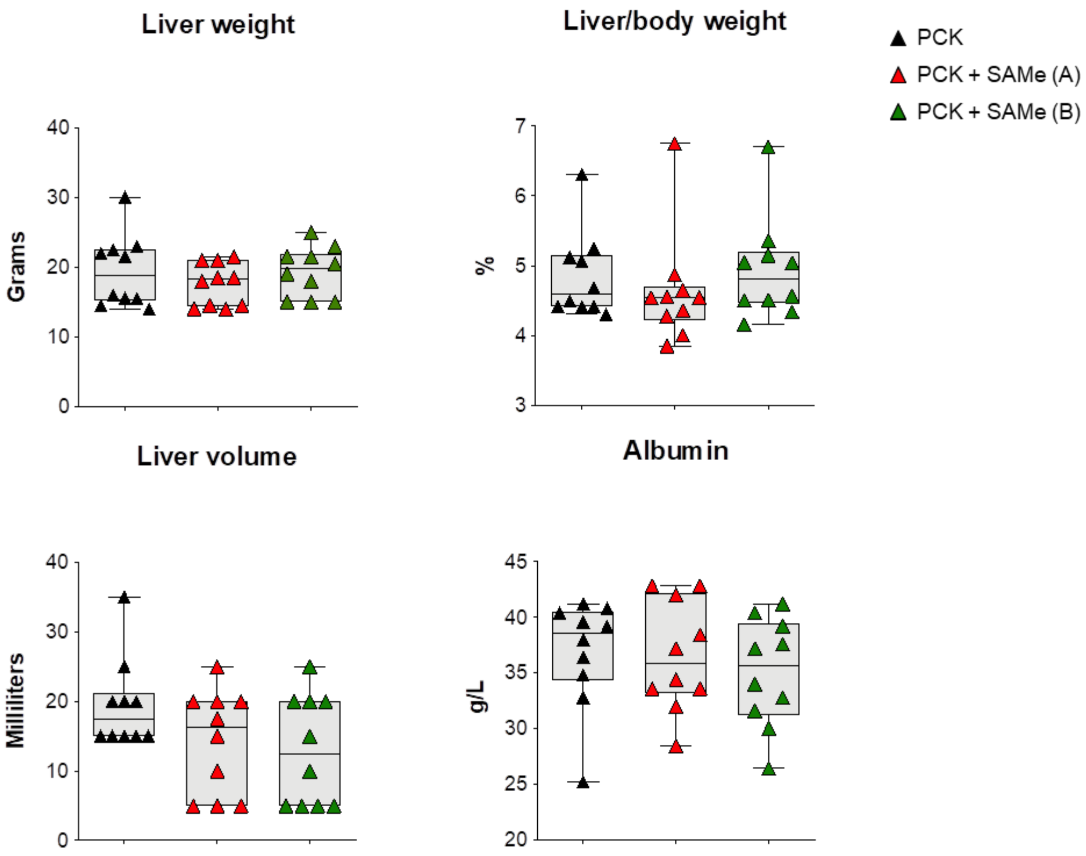

Supplementary Fig. 7. Liver weight, liver/body weight ratio, liver volume and albumin in non-treated PCK and SAMe-treated PCK rats at sacrifice. Two months of SAMe treatment with either (A) 50 mg/kg/day or (B) 100 mg/kg/day. Data shown as mean ± SEM. (one-way ANOVA, Kruskal-Wallis tests)

Supplementary Tables

Supplementary Table 1. Demographic and clinical features of the study cohort.

**Variable**	PLD (n=16)	Gallbladder (n=14)	Healthy liver (n=14)
**Age**, mean ± SEM	51.25 ± 3.07	63.36 ± 3.63	66.36 ± 2.02
**Age**, range	35 – 71	31 – 79	55 - 80
**Gender**			
Female (%)	14 (87.50)	5 (35.71)	7 (50.00)
Male (%)	2 (12.50)	9 (64.29)	7 (50.00)
**Germline mutation**			
PRKCSH (%)			
c.1341-2A>G	4 (25.00)	-	-
c.292+1G>C	1 (6.25)	-	-
SEC63 (%)			
1702delGAA	1 (6.25)	-	-
Other (%)	8 (50.00)	-	-
Unknown (%)	2 (12.50)	-	-
**Number of liver cysts** (%)			
None	0 (0.0)	14 (100.0)	14 (100.0)
<10	2 (12.50)	-	-
10-40	5 (31.25)	-	-
>40	8 (50.00)	-	-
Unknown	1 (6.25)	-	-
**Number of renal cysts** (%)			
None	8 (50.0)	14 (100.0)	14 (100.0)
<10	6 (37.50)	-	-
10-40	1 (6.25)	-	-
>40	1 (6.25)	-	-
**Biochemistry**, median (95% CI)			
ALT (U/L)	27.0 (21.0 – 41.0)	22.0 (12.0 – 102.0)	23.5 (16.0 – 28.0)
AST (U/L)	29.0 (17.0 – 35.0)	23.0 (17.0 – 106.0)	21.5 (17.0 – 30.0)
Albumin (g/L)	38.0 (33.0 – 44.0)	41.8 (40.2 – 50.1)	41.2 (27.6 – 45.7)
Alkaline phosphatase (U/L)	69.0 (61.0 – 119.0)	84.0 (69.0 – 160.0)	69.0 (50.0 – 135.0)
Creatinine (mg/dL)	0.86 (0.72 – 1.18)	0.80 (0.66 – 1.01)	0.80 (0.70 – 1.02)
Blood urea (mg/dL)	29.0 (20.0 – 42.0)	31.5 (25.0 – 52.0)	32.0 (26.0 – 44.0)

Abbreviations: PRKCSH, protein kinase C substrate 80K-H; SEC63, translocation protein SEC63 homolog.

Supplementary Table. 2. Human and rat primers used in qPCR**.**

Primers	Species	Sequence 5' – 3'
ATF6 FW<br>*ATF6* RV	Human	GCTGGATGAAGTTGTGTCAGAG<br>TGTTCCAACATGCTCATAGGTC
ATG5 FW<br>*ATG5* RV	Human	CGTCCTGTGGCTGCAGATG<br>AAGGACAAACTTCTTTGAGGAGA
BAX FW<br>*BAX* RV	Human	CCCGAGAGGTCTTTTTCCGA<br>CCAGCCCATGATGGTTCTGAT
BECN1 FW<br>*BECN1* RV	Human	GATGGAAGGGTCTAAGACGTCCAA<br>TTTCGCCTGGGCTGTGGTAAG
BIM FW<br>*BIM* RV	Human	GCCCCTACCTCCCTACAGAC<br>CCTCATGGAAGCCATTGCAC
CHOP FW<br>*CHOP* RV	Human	TCTTCATACATCACCACACC<br>CTTGTGACCTCTGCTGGTTC
DR5 FW<br>*DR5* RV	Human	ACAGTTGCAGCCGTAGTCTTG<br>CCAGGTCGTTGTGAGCTTCT
GAPDH FW<br>*GAPDH* RV	Human	CCAAGGTCATCCATGACAAC<br>TGTCATACCAGGAAATGAGC
GRP78 FW<br>*GRP78* RV	Human	GAGCTGTGCAGAAACTCCGGCG<br>ACCAGTTGCTGAATCTTTGGAATTCGAGT
IRE1α FW<br>*IRE1α* RV	Human	AGGGAGAGGAGGGAATCGTA<br>CAGTCCCTAATGCCACACCT
LC3B FW<br>*LC3B* RV	Human	GGTGAGAAGCAGCTTCCTGT<br>TCTCCTGGGAGGCATAGACC
MAT1A FW<br>*MAT1A* RV	Human	GTTCACATCGGAGTCTGTGG<br>GATGTGCTTGATGGTGTCC
MAT2A FW<br>*MAT2A* RV	Human	GCTACGAGTAGAACGCTGTC<br>GCATCAGGATCCTGCTGAAG
MAT2B FW<br>*MAT2B* RV	Human	CAGATGCTGCCTCTCAAC<br>GAACCTCTGCTGCCAGTG
SQSTM1/P62 FW<br>*SQSTM1/P62* RV	Human	TACGACTTGTGTAGCGTCTG<br>CGTGTTTCACCTTCCGGAG
PERK FW<br>*PERK* RV	Human	CAGGCAAAGGAAGGAGTCTG<br>AACAACTCCAAAGCCACCAC
SAE1 FW	Human	CTTGCTGCTCCAGGGATGTC

SAE1 RV		GACCATCGTTGTCTCAGAAG
SOX9 FW<br>*SOX9* RV	Human	GTACCCGCACTTGCACAAC<br>TCGCTCTCGTTCAGAAGTCTC
SOX17 FW<br>*SOX17 RV*	Human	GTGGACCGCACGGAATTTG<br>GGAGATTCACACCGGAGTCA
SUMO1 FW<br>*SUMO1* RV	Human	CCGTCATCATGTCTGACC<br>GGAACACCCTGTCTTTGAC
UBA2 FW<br>*UBA2* RV	Human	CGTTGCCTACCATGACAGC<br>GAGGAACATCAGCTGCCAG
UBIQUITIN FW<br>*UBIQUITIN* RV	Human	CCTGAGGGGTGGCTGTTA<br>GCTACCATGCAACGAAACC
UBE2I FW<br>*UBE2I* RV	Human	GGACTTTGAACATGTCGG<br>CCGAAGGGTACACATTCGG
s-XBP1 FW<br>*s-XBP1* RV	Human	GCTGAGTCCGCAGCAGGT<br>CTGGGTCCAAGTTGTCCAGAAT
ZO-1 FW<br>*ZO-1* RV	Human	CGGTCCTCTGAGCCTGTAAG<br>GGATCTACATGCGACGACAA
α-Sma FW<br>*α-Sma* RV	Rat	CGCCATCAGGAACCTCGAGAAG<br>ATCATCACCAGCAAAGCCCG
Col1a1 FW<br>*Col1a1* RV	Rat	GACTGTCCCAACCCCCAAA<br>CTTGGGTCCCTCGACTCCTA
Ctgf FW<br>*Ctgf* RV	Rat	CTAGCTGCCTACCGACTGGA<br>GCCCATCCCACAGGTCTTAG
Cxcl1 (IL8 homolog) FW<br>*Cxcl1 (IL8 homolog)* RV	Rat	ACTCAAGAATGGTCGCGAGG<br>ACGCCATCGGTGCAATCTAT
Gapdh FW<br>*Gapdh* RV	Rat	TGTGAACGGATTTGGCCGTA<br>ATGAAGGGGTCGTTGATGGC
Il6 FW<br>*Il6* RV	Rat	CATTCTGTCTCGAGCCCACC<br>AGTCCCAAGAAGGCAACTGG
Mat1a FW<br>*Mat1a* RV	Rat	CGAGAAGTGTGACACCATG<br>CATCCAGCACTGCATCAC
Mat2a FW<br>*Mat2a* RV	Rat	CACTTCAGAGTCTGTAGG<br>CCTGCACCAATGTCTTCCTC
Mat2b FW<br>*Mat2b* RV	Rat	GAGAAGGAGCTCTCCATCC<br>CAGCAGGTTCACCTGTTCG

Sae1 FW<br>*Sae1* RV	Rat	GTCGACCAGATCTGTCACAG<br>CACTCCAGTCCACTGCTAG
Sumo1 FW<br>*Sumo1* RV	Rat	GACAGCAGTGAGATCCAT<br>CTAAGAGATGGAGTGCCAG
Tgfβ1 FW<br>*Tgfβ1* RV	Rat	CTGCTGACCCCCACTGATAC<br>AGCCCTGTATTCCGTCTCCT
Uba2 FW<br>*Uba2* RV	Rat	CCATGACAGCATCATGAACC<br>CTCTGTCAGGAGACACTTC
Ube2i FW<br>*Ube2i* RV	Rat	CGTGTATCCTTCTGGCAC<br>CTTCGCTTGTGCTCGAAC

Abbreviations: α-Sma, alpha smooth muscle actin; ATF6, activating transcription factor 6; ATG5, autophagy related 5; BAX, Bcl-2 associated X apoptosis regulator; BECN1, Beclin-1; BIM, Bcl-2-like protein 11; CHOP, DNA damage-inducible transcript 3; Col1a1, collagen type 1 alpha 1 chain; Ctgf, connective tissue growth factor; Cxcl1, chemokine (C-X-C motif) ligand 1; DR5, death receptor 5; FW, forward; GAPDH, glyceraldehyde 3-phosphate dehydrogenase; GRP78, heat shock 70kD protein 5; Il6, interleukin 6; IRE1α, serine/threonine-protein kinase/endoribonuclease inositol-requiring enzyme 1α; LC3B, microtubule-associated protein 1 light chain 3 beta; MAT1A, methionine adenosyltransferase 1A; MAT2A; methionine adenosyltransferase 2A; MAT2B; methionine adenosyltransferase 2B; PERK, PKR-like ER kinase; RV, reverse; SAE1, SUMO1 activating enzyme subunit 1; SOX9, SRY-box 9; SOX17, SRY-box 17; SQSTM1, Sequestosome 1; SUMO1, small ubiquitin like modifier 1; s-XBP1, spliced X-box binding protein 1; Tgfβ1, transforming growth factor beta 1; UBA2, SUMO1 activating enzyme subunit 2; UBE2I, ubiquitin conjugating enzyme E2 I; ZO-1, zonula occludens-1.

Supplementary Table. 3. Antibodies used in this study for different applications.

Antibody	Company	Reference	Application
Rabbit monoclonal anti-SUMO1	Abcam	ab32058	IHC, WB
Rabbit monoclonal anti-SUMO1 (BSA and Azide free)	Abcam	ab219724	IP, WB
Rabbit monoclonal anti-UBC9	Abcam	ab75854	IHC, WB
Rabbit monoclonal anti-Ubiquitin	Abcam	ab134953	IHC, WB
Mouse monoclonal anti-p62	Abcam	ab56416	IHC, WB
Mouse monoclonal anti-Acetylated α-tubulin	Sigma-Aldrich	T6793	IF, WB
Rabbit monoclonal anti- α-tubulin	Abcam	ab52866	IF, WB
Mouse monoclonal anti β-actin	Sigma	A5316	WB
Rabbit monoclonal IgG	Abcam	ab172730	IP
Anti-mouse IgG, HRP-linked Antibody	Cell Signaling	#7076	WB
Anti-rabbit IgG, HRP-linked Antibody	Cell Signaling	#7074	WB
Anti-mouse IgG, Alexa Fluor 488	Invitrogen	A28175	IF

Abbreviations: HRP, horseradish peroxidase; IgG, immunoglobulin G; IP, immunoprecipitation; IHC, immunohistochemistry; SUMO1, small ubiquitin-related modifier 1; UBC9, SUMO-conjugating enzyme UBC9; WB, Western Blot.

Supplementary Table. 4. Physical and serum biochemical parameters of the animals at sacrifice.

	A	*B*	*C*			
**Parameters (units)**	**Wild-type Rats**	**PCK Rats (non-treated)**	**PCK Rats (SAMe-treated)**	**p value (*A* vs *B*)**	**p value (*B* vs *C*)**	**p value (*C* vs *A*)**
**Body weight (g)**	542.3 ± 64.09	486.2 ± 33.99	459.5 ± 31.67	0.403	0.576	0.217
**Kidney weight (g)**	2.61 ± 0.27	4.06 ± 0.56	3.28 ± 0.42	0.078	0.293	0.254
**Kidney/body weight (%)**	0.49 ± 0.02	0.88 ± 0.16	0.73 ± 0.11	<0.0001*	0.252	0.0002*
**Bile flow (µl/min/g)**	0.026 ± 0.003	0.047 ± 0.010	0.025 ± 0.006	0.111	0.110	0.481
**Alkaline phosphatase (U/L)**	71.5 ± 7.50	187.4 ± 16.85	158.7 ± 13.31	<0.0001*	0.199	<0.0001*
**Aspartate aminotransferase (U/L)**	233.0 ± 54.48	243.1 ± 22.33	234.7 ± 23.06	0.842	0.795	0.975
**Alanine aminotransferase (U/L)**	46.50 ± 4.95	44.29 ± 4.53	47.67 ± 2.80	0.769	0.097	0.828
**Total protein (g/dL)**	6.51 ± 0.41	6.36 ± 0.23	6.37 ± 0.13	0.736	0.970	0.716
**Blood urea (mg/dL)**	39.50 ± 2.20	36.92 ± 2.72	35.00 ± 2.75	0.516	0.462	0.016*

* = significant

Values as Mean + SEM. *p<0.05. (t-tests) *Abbreviations*: SAMe, S-adenosylmethionine

Supplementary Table 5. Differential proteomic profile of shRNA-*UBE2I* PHC compared to control and shRNA-control PHC.

UniProt Accession number	Protein	p value	Abundancy
Q6L8Q7	2' 5'-phosphodiesterase 12 (PDE12)	0.01	Increased
O15530	3-phosphoinositide-dependent protein kinase 1 (PDPK1)	0.02	Increased
Q9H0D6	5'-3' exoribonuclease 2 (XRN2)	0.05	Increased
Q04917	14-3-3 protein eta (YWHAH)	0.02	Increased
P05386	60S acidic ribosomal protein P1 (RPLP1)	0.03	Decreased
P83881	60S ribosomal protein L36a (RPL36A)	0.01	Increased
P68133	Actin. alpha skeletal muscle (ACTA1)	0.03	Increased
Q10588	ADP-ribosyl cyclase/cyclic ADP-ribose hydrolase 2 (BST1)	0.02	Increased
Q04828	Aldo-keto reductase family 1 member C1 (AKR1C1)	0.001	Decreased
Q9UKV3	Apoptotic chromatin condensation inducer in the nucleus (ACIN1)	0.04	Increased
Q15121	Astrocytic phosphoprotein PEA-15 (PEA15)	0.0003	Increased
Q9UHQ4	B-cell receptor-associated protein 29 (BCAP29)	0.04	Increased
P54687	Branched-chain-amino-acid aminotransferase, cytosolic (BCAT1)	0.03	Increased
P19022	Cadherin-2 (CDH2)	0.03	Increased
Q05682	Caldesmon (CALD1)	0.01	Increased
P07384	Calpain-1 catalytic subunit (CAPN1)	0.01	Increased
P13861	cAMP-dependent protein kinase type II-alpha regulatory subunit (PRKAR2A)	0.02	Decreased
P23786	Carnitine O-palmitoyltransferase 2, mitocondrial (CPT2)	0.00	Increased
P30622	CAP-Gly domain-containing linker protein 1 (CLIP1)	0.001	Increased
P43155	Carnitine O-acetyltransferase (CRAT)	0.02	Increased
Q9P000	COMM domain-containing protein 9 (COMMD9)	0.01	Increased
P14384	Carboxypeptidase M (CPM)	0.04	Increased
Q9NWV4	CXXC motif containing zinc binding protein (CZIB)	0.03	Increased
Q96LJ7	Dehydrogenase/reductase SDR family member 1 (DHRS1)	0.04	Increased
P09622	Dihydrolipoyl dehydrogenase, mitochondrial (DLD)	0.04	Increased
O15160	DNA-directed RNA polymerases I and III subunit RPAC1 (POLR1C)	0.04	Increased
P04844	Dolichyl-diphosphooligosaccharide—protein glycosyltransferase subunit 2 (RPN2)	0.0001	Decreased
Q96FJ2	Dynein light chain 2, cytoplasmic (DYNLL2)	0.05	Increased
Q63HN8	E3 ubiquitin-protein ligase RNF213 (RNF213)	0.02	Increased
Q9H4M9	EH domain-containing protein 1 (EHD1)	0.03	Increased

Q8IUD2	ELKS/Rab6-interacting/CAST family member 1 (ERC1)	0.04	Increased
P13639	Elongation factor 2 (EEF2)	0.02	Decreased
Q9UKM7	Endoplasmic reticulum mannosyl-oligosaccharide 1 2-alpha-mannosidase (MAN1B1)	0.02	Increased
O43414	ERI1 exoribonuclease 3 (ERI3)	0.05	Increased
Q8NBQ5	Estradiol 17-beta-dehydrogenase 11 (HSD17B11)	0.01	Increased
P23588	Eukaryotic translation initiation factor 4B (EIF4B)	0.04	Increased
P42285	Exosome RNA helicase MTR4 (MTREX)	0.04	Decreased
Q16610	Extracellular matrix protein 1 (ECM1)	0.04	Increased
P35555	Fibrillin-1 (FBN1)	0.01	Increased
Q8WUP2	Filamin-binding LIM protein 1 (FBLIM1)	0.03	Increased
P48506	Glutamate--cysteine ligase catalytic subunit (GCLC)	0.04	Increased
P35754	Glutaredoxin-1 (GLRX)	0.0002	Increased
P35573	Glycogen debranching enzyme (AGL)	0.01	Increased
Q8TBA6	Golgin subfamily A member 5 (GOLGA5)	0.01	Increased
Q96CP6	GRAM domain-containing protein 1A (GRAMD1A)	0.01	Increased
Q15477	Helicase SKI2W (SKIV2L)	0.04	Increased
P52926	High mobility group protein HMGI-C (HMGA2)	0.04	Increased
P68431	Histone H3.1 (HIST1H3A)	0.04	Decreased
Q71DI3	Histone H3.2 (HIST2H3A)	0.01	Decreased
Q86X55	Histone-arginine methyltransferase CARM1 (CARM1)	0.03	Increased
Q16543	Hsp90 co-chaperone Cdc37 (CDC37)	0.04	Decreased
Q16836	Hydroxyacyl-coenzyme A dehydrogenase, mitochondrial (HADH)	0.04	Increased
Q9UI26	Importin-11 (IPO11)	0.04	Increased
Q15323	Keratin, type I cuticular Ha1 (KRT31)	0.05	Increased
Q16850	Lanosterol 14-alpha demethylase (CYP51A1)	0.05	Decreased
P46379	Large proline-rich protein BAG6 (BAG6)	0.01	Decreased
Q14847	LIM and SH3 domain protein 1 (LASP1)	0.01	Decreased
Q86W92	Liprin-beta-1 (PPFIBP1)	0.01	Increased
P36776	Lon protease homolog, mitochondrial (LONP1)	0.0003	Decreased
P38571	Lysosomal acid lipase/cholesteryl ester hydrolase (LIPA)	0.003	Increased
Q66K74	Microtubule-associated protein 1S (MAP1S)	0.01	Decreased
O95819	Mitogen-activated protein kinase kinase kinase kinase 4 (MAP4K4)	0.04	Increased
P35749	Myosin-11 (MYH11)	0.04	Decreased
P19105	Myosin regulatory light chain 12A (MYL12A)	0.01	Increased
P48681	Nestin (NES)	0.0001	Increased
P19338	Nucleolin (NCL)	0.003	Decreased
Q01085	Nucleolysin TIAR (TIAL1)	0.03	Decreased
Q96EE3	Nucleoporin SEH1 (SEH1L)	0.05	Increased

Q96RS6	NudC domain-containing protein 1 (NUDCD1)	0.04	Decreased
Q96CV9	Optineurin (OPTN)	0.03	Increased
P20962	Parathymosin (PTMS)	0.01	Increased
Q96AY3	Peptidyl-prolyl cis-trans isomerase FKBP10 (FKBP10)	0.00001	Decreased
Q99541	Perilipin-2 (PLIN2)	0.04	Increased
Q14558	Phosphoribosyl pyrophosphate synthase-associated protein 1 (PRPSAP1)	0.02	Decreased
Q86UU1	Pleckstrin homology-like domain family B member 1 (PHLDB1)	0.04	Increased
P15151	Poliovirus receptor (PVR)	0.04	Increased
Q9UKK3	Poly [ADP-ribose] polymerase 4 (PARP4)	0.02	Increased
P51531	Probable global transcription activator SNF2L2 (SMARCA2)	0.05	Decreased
P07602	Prosaposin (PSAP)	0.02	Decreased
P25788	Proteasome subunit alpha type-3 (PSMA3)	0.02	Increased
Q8N8S7	Protein enabled homolog (ENAH)	0.02	Increased
Q9BRX2	Protein pelota homolog (PELO)	0.01	Increased
P29590	Protein PML (PML)	0.01	Increased
Q99584	Protein S100-A13 (S100A13)	0.02	Decreased
Q9Y2Z0	Protein SGT1 homolog (SUGT1)	0.04	Decreased
P14618	Pyruvate kinase PKM (PKM)	0.03	Increased
Q9Y3P9	Rab GTPase-activating protein 1 (RABGAP1)	0.02	Increased
P62820	Ras-related protein Rab-1A (RAB1A)	0.03	Decreased
P61006	Ras-related protein Rab-8A (RAB8A)	0.03	Increased
P11233	Ras-related protein Ral-A (RALA)	0.03	Increased
Q8N122	Regulatory-associated protein of mTOR (RPTOR)	0.02	Increased
Q16799	Reticulon-1 (RTN1)	0.005	Increased
Q52LW3	Rho GTPase-activating protein 29 (ARHGAP29)	0.001	Increased
Q92974	Rho guanine nucleotide exchange factor 2 (ARHGEF2)	0.02	Increased
Q15052	Rho guanine nucleotide exchange factor 6 (ARHGEF6)	0.03	Increased
P08134	Rho-related GTP-binding protein RhoC (RHOC)	0.02	Increased
O94804	Serine/threonine-protein kinase 10 (STK10)	0.03	Increased
Q9H788	SH2 domain-containing protein 4A (SH2D4A)	0.02	Increased
Q9Y512	Sorting and assembly machinery component 50 homolog (SAMM50)	0.05	Increased
Q12874	Splicing factor 3A subunit 3 (SF3A3)	0.03	Increased
Q8NBJ7	Sulfatase-modifying factor 2 (SUMF2)	0.01	Decreased
Q92922	SWI/SNF complex subunit SMARCC1 (SMARCC1)	0.01	Increased
Q9UH65	Switch-associated protein 70 (SWAP70)	0.01	Increased
Q9UMS6	Synaptopodin-2 (SYNPO2)	0.01	Increased
Q66K14	TBC1 domain family member 9B (TBC1D9B)	0.04	Increased

Q9NUJ3	T-complex protein 11-like protein 1 (TCP11L1)	0.03	Increased
Q9UGI8	Testin (TES)	0.03	Increased
Q5JTV8	Torsin-1A-interacting protein 1 (TOR1AIP1)	0.01	Increased
P37802	Transgelin-2 (TAGLN2)	0.03	Increased
Q99442	Translocation protein SEC62 (SEC62)	0.03	Increased
Q15363	Transmembrane emp24 domain-containing protein 2 (TMED2)	0.02	Decreased
P07951	Tropomyosin beta chain (TPM2)	0.05	Increased
Q15628	Tumor necrosis factor receptor type 1-associated DEATH domain protein (TRADD)	0.02	Increased
P78324	Tyrosine-protein phosphatase non-receptor type substrate 1 (SIRPA)	0.04	Increased
Q14376	UDP-glucose 4-epimerase (GALE)	0.05	Increased
P15374	Ubiquitin carboxyl-terminal hydrolase isozyme L3 (UCHL3)	0.04	Increased
O43795	Unconventional myosin-Ib (MYO1B)	0.03	Increased
Q9NP79	Vacuolar protein sorting-associated protein VTA1 homolog (VTA1)	0.003	Decreased
Q2TAY7	WD40 repeat-containing protein SMU1 (SMU1)	0.03	Decreased

Abbreviations: PHC, polycystic human cholangiocytes; shRNA, short hairpin RNA; UBE2I, Ubiquitin conjugating enzyme E2 I.

Supplementary Table 6. Differential SUMOylated proteins identified after SUMO1-IP in PHC compared to NHC.

UniProt Accession number	Protein	p value	Abundancy
P35998	26S protease regulatory subunit 7 (PSMC2)	0.0054	Increased
P62241	40S ribosomal protein S8 (RPS8)	0.0495	Decreased
P26373	60S ribosomal protein L13 (GN=RPL13)	0.0008	Increased
P18621	60S ribosomal protein L17 (RPL17)	0.0150	Increased
P62424	60S ribosomal protein L7a (RPL7A)	0.0014	Increased
P61158	Actin-related protein 3 (ACTR3)	0.0097	Increased
Q12797	Aspartyl/asparaginyl beta-hydroxylase (ASPH)	0.0388	Increased
Q9Y696	Chloride intracellular channel protein 4 (CLIC4)	0.0052	Increased
Q9Y678	Coatomer subunit gamma-1 (COPG1)	0.0097	Increased
P60981	Destrin (DSTN)	0.0043	Decreased
Q9UJU6	Drebrin-like protein (DBNL)	0.0367	Increased
O60763	General vesicular transport factor p115 (USO1)	0.0015	Increased
P11413	Glucose-6-phosphate 1-dehydrogenase (G6PD)	0.0009	Increased
P00367	Glutamate dehydrogenase 1, mitochondrial (GLUD1)	0.0496	Increased
P41250	Glycine--tRNA ligase (GARS)	0.0472	Increased
P28799	Granulins (GRN)	0.0004	Increased
P62826	GTP-binding nuclear protein Ran (RAN)	0.0006	Increased
P04259	Keratin, type II cytoskeletal 6B (KRT6B)	0.00002	Decreased
Q9BS40	Latexin (LXN)	0.0253	Increased
P00387	NADH-cytochrome b5 reductase 3 (CYB5R3)	0.0008	Increased
P12273	Prolactin-inducible protein (PIP)	0.0436	Increased
Q32P28	Prolyl 3-hydroxylase 1 (P3H1)	0.0416	Increased
P31151	Protein S100-A7 (S100A7)	0.0005	Decreased
P46060	Ran GTPase-activating protein 1 (RANGAP1)	0.0002	Increased
Q9H0U4	Ras-related protein Rab-1B (RAB1B)	0.0227	Increased
Q9UJZ1	Stomatin-like protein 2, mitochondrial (STOML2)	0.0005	Increased

Abbreviations: IP, immunoprecipitation; NHC, normal human cholangiocytes; PHC, polycystic human cholangiocytes.

Supplementary Table 7. Physical and serum biochemical parameters of the animals at sacrifice. Two months of SAMe treatment with either (B) 50 mg/kg/day or (C) 100 mg/kg/day.

	A	***B***	***C***		
**Parameters (units)**	**PCK rats**	**PCK Rats (50 mg SAMe-treated)**	**PCK Rats (100 mg SAMe-treated)**	**p value (*A* vs *B*)**	**p value (*C* vs *A*)**
**Body weight (g)**	402.4 ± 30.78	384.9 ± 81.62	402.9 ± 34.88	0.393	0.684
**Kidney weight (g)**	2.47 ± 0.17	2.43 ± 0.14	2.60 ± 0.17	0.854	0.591
**Kidney/body weight (%)**	0.62 ± 0.01	0.64 ± 0.02	0.67 ± 0.05	0.401	0.347
**Bile flow (µl/min/g)**	0.029 ± 0.006	0.031 ± 0.008	0.022 ± 0.003	0.435	0.166
**Alkaline phosphatase (U/L)**	186.8 ± 14.99	182.0 ± 18.19	196.8 ± 11.18	0.841	0.599
**Aspartate aminotransferase (U/L)**	316.0 ± 32.91	332.4 ± 23.95	241.2 ± 18.18	0.591	0.031*+
**Alanine aminotransferase (U/L)**	46.00 ± 3.64	46.40 ± 1.71	41.60 ± 2.47	0.176	0.361
**Total protein (g/dL)**	6.08 ± 0.11	6.04 ± 0.19	5.89 ± 0.16	0.885	0.360
**Blood urea (mg/dL)**	35.60 ± 1.11	32.80 ± 0.80	38.00 ± 1.37	0.028*+	0.189

* = significant; + = one-tailed *t*-test.

Values as Mean + SEM. *Abbreviations*: SAMe, S-adenosylmethionine

References

[1] **Erice O**, **Munoz-Garrido P**, Vaquero J, Perugorria MJ, Fernandez-Barrena MG, Saez E, et al. MicroRNA-506 promotes primary biliary cholangitis-like features in cholangiocytes and immune activation. Hepatology 2018;67:1420–40. https://doi.org/10.1002/hep.29533.

[2] **Ward CJ**, Hogan MC, Rossetti S, Walker D, Sneddon T, Wang X, et al. The gene mutated in autosomal recessive polycystic kidney disease encodes a large, receptor-like protein. Nat Genet 2002;30:259–69. https://doi.org/10.1038/ng833.

[3] **Mason SB**, Liang Y, Sinders RM, Miller CA, Eggleston-Gulyas T, Crisler-Roberts R, et al. Disease Stage Characterization of Hepatorenal Fibrocystic Pathology in the PCK Rat Model of ARPKD. Anat Rec Adv Integr Anat Evol Biol 2010;293:1279–88. https://doi.org/10.1002/ar.21166.

[4] **Masyuk T V.**, Huang BQ, Masyuk AI, Ritman EL, Torres VE, Wang X, et al. Biliary Dysgenesis in the PCK Rat, an Orthologous Model of Autosomal Recessive Polycystic Kidney Disease. Am J Pathol 2004;165:1719. https://doi.org/10.1016/S0002-9440(10)63427-X.

[5] **Masyuk T V,** Masyuk AI, Lorenzo Pisarello M, Howard BN, Huang BQ, Lee P-Y, et al. TGR5 contributes to hepatic cystogenesis in rodents with polycystic liver diseases through cyclic adenosine monophosphate/Gαs signaling. Hepatology 2017;66:1197–218. https://doi.org/10.1002/hep.29284.

[6] **Masyuk T V**, Radtke BN, Stroope AJ, Banales JM, Gradilone SA, Huang B, et al. Pasireotide is more effective than octreotide in reducing hepatorenal cystogenesis in rodents with polycystic kidney and liver

diseases. Hepatology 2013;58:409–21. https://doi.org/10.1002/hep.26140.

[7] **Masyuk T V.**, Masyuk AI, Torres VE, Harris PC, Larusso NF. Octreotide Inhibits Hepatic Cystogenesis in a Rodent Model of Polycystic Liver Disease by Reducing Cholangiocyte Adenosine 3′,5′-Cyclic Monophosphate. Gastroenterology 2007;132:1104–16. https://doi.org/10.1053/j.gastro.2006.12.039.

[8] **Lorenzo Pisarello M**, Masyuk TV, Gradilone SA, Masyuk AI, Ding JF, Lee P-Y, et al. Combination of a Histone Deacetylase 6 Inhibitor and a Somatostatin Receptor Agonist Synergistically Reduces Hepatorenal Cystogenesis in an Animal Model of Polycystic Liver Disease. Am J Pathol 2018;188. https://doi.org/10.1016/j.ajpath.2017.12.016.

[9] **Wunsch E**, Raszeja-Wyszomirska J, Barbier O, Milkiewicz M, Krawczyk M, Milkiewicz P. Effect of S-adenosyl-L-methionine on liver biochemistry and quality of life in patients with primary biliary cholangitis treated with ursodeoxycholic acid. A prospective, open label pilot study. J Gastrointest Liver Dis 2018;27:273–9. https://doi.org/10.15403/jgld.2014.1121.273.icz.

[10] **Mato JM,** Cámara J, Fernández de Paz J, Caballería L, Coll S, Caballero A, et al. S-adenosylmethionine in alcoholic liver cirrhosis: a randomized, placebo-controlled, double-blind, multicenter clinical trial. J Hepatol 1999;30:1081–9.

[11] **Schneider CA**, Rasband WS, Eliceiri KW. NIH Image to ImageJ: 25 years of image analysis. Nat Methods 2012;9:671–5. https://doi.org/10.1038/nmeth.2089.

[12] **Xiao Y,** Pollack D, Nieves E, Winchell A, Callaway M, Vigodner M. Can your protein be sumoylated? A quick summary and important tips to study

SUMO-modified proteins. Anal Biochem 2015;477:95–7. https://doi.org/10.1016/j.ab.2014.11.006.

[13] **Salter KD**, Roman RM, LaRusso NR, Fitz JG, Doctor RB. Modified Culture Conditions Enhance Expression of Differentiated Phenotypic Properties of Normal Rat Cholangiocytes. Lab Investig 2000;80:1775–8. https://doi.org/10.1038/labinvest.3780187.

[14] **Wiśniewski JR**, Zougman A, Nagaraj N, Mann M. Universal sample preparation method for proteome analysis. Nat Methods 2009;6:359–62. https://doi.org/10.1038/nmeth.1322.

[15] **Meier F**, Beck S, Grassl N, Lubeck M, Park MA, Raether O, et al. Parallel Accumulation–Serial Fragmentation (PASEF): Multiplying Sequencing Speed and Sensitivity by Synchronized Scans in a Trapped Ion Mobility Device. J Proteome Res 2015;14:5378–87. https://doi.org/10.1021/acs.jproteome.5b00932.

[16] **Meier F**, Brunner A-D, Koch S, Koch H, Lubeck M, Krause M, et al. Online Parallel Accumulation–Serial Fragmentation (PASEF) with a Novel Trapped Ion Mobility Mass Spectrometer. Mol Cell Proteomics 2018;17:2534–45. https://doi.org/10.1074/mcp.TIR118.000900.

[17] **Tyanova S**, Temu T, Sinitcyn P, Carlson A, Hein MY, Geiger T, et al. The Perseus computational platform for comprehensive analysis of (prote)omics data. Nat Methods 2016;13:731–40. https://doi.org/10.1038/nmeth.3901.

[18] **Szklarczyk D**, Gable AL, Lyon D, Junge A, Wyder S, Huerta-Cepas J, et al. STRING v11: protein–protein association networks with increased coverage, supporting functional discovery in genome-wide experimental

datasets. Nucleic Acids Res 2019;47:D607--D613. https://doi.org/10.1093/nar/gky1131.

[19] **Huang DW**, Sherman BT, Lempicki RA. Systematic and integrative analysis of large gene lists using DAVID bioinformatics resources. Nat Protoc 2009;4:44–57. https://doi.org/10.1038/nprot.2008.211.

[20] **Babicki S**, Arndt D, Marcu A, Liang Y, Grant JR, Maciejewski A, et al. Heatmapper: web-enabled heat mapping for all. Nucleic Acids Res 2016;44:W147-53. https://doi.org/10.1093/nar/gkw419.

[21] **Perez-Riverol Y**, Csordas A, Bai J, Bernal-Llinares M, Hewapathirana S, Kundu DJ, et al. The PRIDE database and related tools and resources in 2019: improving support for quantification data. Nucleic Acids Res 2019;47:D442–50. https://doi.org/10.1093/nar/gky1106.
